# HPLC- and NMR-Based Chemical Profiling, Wound-Healing Potential, Anti-Inflammatory and Antibacterial Activities of *Satureja pilosa* (Lamiaceae), a Neglected Medicinal–Aromatic Herb

**DOI:** 10.3390/plants12244114

**Published:** 2023-12-08

**Authors:** Christina Panagiotidou, Luisa D. Burgers, Christina Tsadila, Chara Almpani, Nikos Krigas, Dimitris Mossialos, Michail Christou Rallis, Robert Fürst, Anastasia Karioti

**Affiliations:** 1 Laboratory of Pharmacognosy, School of Pharmacy, Aristotle University of Thessaloniki, University Campus, 54124 Thessaloniki, Greece; cristinapanagiotidou@hotmail.com; 2 Institute of Pharmaceutical Biology, Faculty of Biochemistry, Chemistry and Pharmacy, Goethe University, 60438 Frankfurt, Germany; burgers@em.uni-frankfurt.de (L.D.B.); fuerst@em.uni-frankfurt.de (R.F.); 3 Microbial Biotechnology-Molecular Bacteriology-Virology Laboratory, Department of Biochemistry & Biotechnology, University of Thessaly, 41500 Larissa, Greece; tsadila@uth.gr (C.T.); mosial@bio.uth.gr (D.M.); 4 Department of Pharmaceutical Technology, School of Pharmacy, National & Kapodistrian University of Athens, Panepistimiopolis, Zografou, 15784 Athens, Greece; ch.almpani@gmail.com (C.A.); rallis@pharm.uoa.gr (M.C.R.); 5 Hellenic Agricultural Organization—Demeter (ELGO DIMITRA), Institute of Breeding and Plant Genetic Resources, 57001 Thermi, Greece; nikoskrigas@gmail.com; 6 LOEWE Center for Translational Biodiversity Genomics (LOEWE-TBG), 60325 Frankfurt, Germany

**Keywords:** NIH/3T3 fibroblasts, endothelial cells, inflammation, angiogenesis, antibacterial ac-tivity, winter savory, *Satureja pilosa*, clinopodic acids

## Abstract

*Satureja pilosa* Velen. (Lamiaceae) is a perennial and melliferous aromatic–medicinal subshrub which is range-restricted in adjacent parts of Greece and Bulgaria and locally in Italy, known in Northern Greece as wild oregano (“agriorigani”) and traditionally collected from the wild for culinary purposes. Since the ethnopharmacological data and modern biological activities of *Satureja* spp. suggest promising applications in skin conditions, the present study aimed to investigate the hitherto unknown phenolic content of cultivated *S. pilosa* and its potential biological activities, focusing mainly on wound-healing and anti-inflammatory effects. An HPLC-PDA-MS-targeted phytochemical investigation, along with NMR, allowed for the isolation and characterization of the main constituents, resulting in 18 compounds. Representative extracts and purified compounds were tested for wound-healing activity on NIH/3T3 fibroblasts. The butanol extract exhibited a significantly higher cell migration rate (73.4%) compared to aqueous (50.6%) and methanolic (49.6%) ones, enhancing the cell migration more rapidly at both concentration levels, whilst rosmarinic acid was the most potent among the isolated compounds, with a migration rate of 64.0% at the concentration level of 10^−5^ mg/mL, followed by 3,4-dihydrophenyllactic acid (54.7%). Moreover, potential effects on endothelial activation processes were explored, including the leukocyte–endothelial cell interaction during inflammatory processes and the migratory capacity during angiogenic actions, since these processes are commonly associated with skin diseases. Finally, extracts and purified compounds demonstrated weak antibacterial potential against two important pathogens (*Staphylococcus aureus* and *Pseudomonas aeruginosa*), suggesting that further investigation is warrented.

## 1. Introduction

The genus *Satureja* (Lamiaceae) comprises more than 40 medicinal–aromatic plant species distributed mainly in the Mediterranean region [1]. Early reports of such plants under the ancient name “thymbra” seem to date back to Hippocrates times, although a detailed botanical description was only provided later by Theophrastus [2]. The name “satureia” (herb of satyrs) is originally attributed to Pliny, whereas the common name “savory” for some members of this genus is linked to the aromatic and carminative properties of these plants, rendering them important culinary herbs, e.g., winter and summer savory [3].

In addition to their pleasant organoleptic properties, *Satureja* spp. are medicinal plants that have been traditionally used as muscle-pain-relievers, tonics, and carminative agents, or to treat stomach and intestinal disorders such as cramps, nausea, indigestion, diarrhea, and acute gastrointestinal enteritis [4,5]. The members of this genus are known for their antimicrobial and antioxidant essential oils, which are rich in carvacrol, thymol and linalool, as well as for a wide range of antioxidant phenolics including flavonoids and depsides. The main representatives of the non-volatile fraction are usually rosmarinic acid and oligomeric depsides as derivatives of caffeic and 3,4-dihydroxyphenylactic acid [3,4]. These compounds are responsible for the tannic and astringent effect of *Satureja* spp. and, along with the antiseptic properties of the volatile content, they contribute to resolving diarrhea and gastroenteritis events [5,6].

Topically, *Satureja* spp. have been used both in the form of essential oils and as extracts to treat several skin conditions. The essential oils are often used in skincare products for acne and psoriasis [7] due to their aforementioned antimicrobial effect. Due to their delicate perfume, especially when they have a high linalool content, *Satureja* spp. are also used in perfumes and soaps. For example, the essential oil of *S. montana* L., which is rich in geraniol and trans-*β*-caryophyllene, is reported to be efficient in the prevention and treatment of grease-prone skin and the associated aesthetic skin defects caused by *Cutibacterium acnes* (formerly *Propionibacterium acnes)* [8]. In Italy, the aqueous extracts of some *Satureja* spp. are used traditionally in combination with other aromatic plants, such as lavender, rosemary and St. John’s wort, as skin toners [7]. In the Balkan Peninsula, extracts of *S*. *hortensis* L. and *S. montana* are used for wounds, whereas the local Balkan endemic *S*. *kitaibelii* Wierzb. ex Heuff. is reported to have analgesic properties and is used for the treatment of skin infections and inflammations [9]. Recently, extracts of the latter species have been reported to be effective against Fem-x melanoma cells, showing strong antitumor activity [10].

Among other skin conditions, wound-healing is a complex and well-coordinated process, with numerous factors being activated or inhibited in a cascade of events in three successive but overlapping phases, namely inflammation, proliferation and tissue formation [11,12,13]. In general, the objective of any wound management is to heal wounds as rapidly as possible with minimal pain, discomfort, and scarring. To this end, numerous medicinal plants and natural products thereof, such as *Achillea* spp., *Hamamelis virginiana* L., *Echinacea purpurea* (L.) Moench, *Hyperici oleum*, betulin and others, are traditionally used for wound-healing as they exert anti-inflammatory, anti-microbial, antioxidant and regenerating properties [14,15,16,17,18,19] favoring the wound-healing process. The latter includes the activation of fibroblasts, as well as other endothelial cells which play a crucial role in wound-healing, and their activation is needed to prevent the formation of chronic wounds [20]. Fibroblasts (main component of dermis) participate in the process of wound-healing by releasing tropocollagen and tropoelastin as precursors of the elastic skin fibers. Endothelial cells build the inner lining of all blood vessels, including the microvasculature of the dermis. During wound-healing, endothelial cells participate in the formation of new blood vessels (angiogenesis) to recreate the vascular network after tissue injury [21].

However, and in contrast to their beneficial actions during wound-healing, when fibroblasts, endothelial cells and cells of the peripheral immune system are excessively activated during wound-healing, they may also enhance the onset of several skin diseases (including psoriasis) through excessive angiogenesis and inflammation [22,23,24]. Hence, therapeutic compounds with anti-angiogenic and anti-inflammatory actions might also be useful in the treatment of skin infections and diseases.

In general, plant extracts that are rich in antioxidant constituents are efficient agents, limiting the reactive oxygen species (ROS) that can induce photoaging, and may also hinder inflammatory processes in the wound-healing process, thus enabling fibroblasts to restore. For example, a previous phytochemical study on wild-growing *Satureja pilosa* Velen. [25] has shown that it contains a remarkable amount of antioxidant compounds, especially rosmarinic acid and the rare clinopodic acids, as well as various flavonoids, mainly luteolin and eriodictyol derivatives [26,27,28].

From another viewpoint, all *Satureja* spp., which have been praised since Virgil times for their development of aromatic honeys [29], are well-known melliferous species, which can also be exploited as ornamental aromatic–medicinal plants for culinary purposes or gardening and landscape applications, and therefore, they are studied regarding their potential for cultivation and sustainable exploitation [30,31,32]. Among 15 wild-growing *Satureja* species and subspecies in Greece [33], *S. pilosa* is a perennial subshrub, which is range-restricted in adjacent parts of Greece and Bulgaria and locally in Italy. Due to its distinctive and delicate lemon–oregano or lemon–thyme scent [34], *S. pilosa* is known locally in Northern Greece as wild oregano (“agriorigani”) and is traditionally collected from the wild for culinary and medicinal purposes.

In this context, the aim of this work was to access the phenolic content of the cultivated *S. pilosa* and evaluate its potential application in skin ailments. To this end, the *S. pilosa* polar extract was subjected to chromatographic analyses and the main fractions were characterized qualitatively and quantitatively by HPLC-PDA-MS. Then, we tested both fractions and purified constituents thereof for wound-healing activity on NIH/3T3 fibroblasts. Further effects on angiogenic actions as well as on inflammatory processes were tested in primary human umbilical vein endothelial cells (HUVECs). Lastly, the antimicrobial effect of major compounds and extracts was tested against *Pseudomonas aeruginosa* and *Staphylococcus aureus,* which are important nosocomial pathogens that often impede the wound-healing process [35,36].

## 2. Results

In the present work, a cultivated sample of *S. pilosa* was assessed for the first time for its chemical content and evaluated for in vitro biological effects related to skin conditions. The phytochemical analysis was performed in two directions: an HPLC-PDA-MS characterization of selected extracts/fractions, and the chromatographic isolation of major constituents thereof. The biological effects of both extracts and the main isolated metabolites examined the wound-healing, anti-inflammatory and antibacterial activities.

### 2.1. Isolation of the Main/Characteristic Constituents

**Figure 1:**
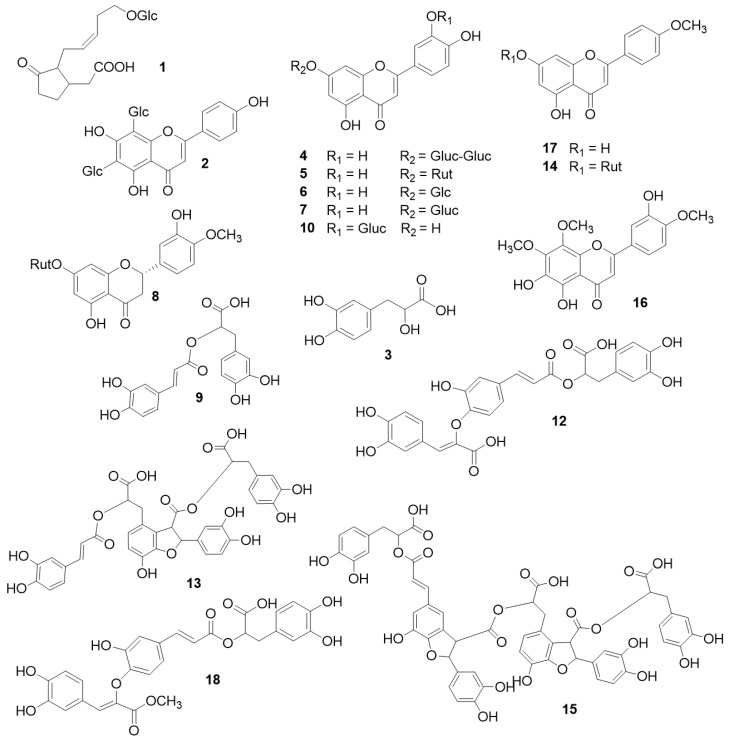
Constituents isolated and/or detected in *S. pilosa* methanol extract. Glc: glucose; Gluc: glucuronic acid; Rut: rutinoside (rhamnosyl 1→6-glucoside); 12-hydroxyjasmonic acid glucoside (**1**); vicenin 2 (**2**); 3,4-dihydroxyphenyllactic acid (**3**); luteolin 7-*O*-diglucuronide (**4**); luteolin 7-O-rutinoside (**5**); luteolin 7-*O*-glucoside (**6**); luteolin 7-*O*-glucuronide (**7**); hesperidin (hesperitin 7-*O*-rutinoside) (**8**); rosmarinic acid (**9**); luteolin 3′-*O*-glucuronide (**10**); melitric acid A (**12**); clinopodic acid I (**13**); acacetin 7-*O*-rhamnosylglucoside (**14**); clinopodic acid O (**15**); 5,6,3-trihydroxy-7,8,4-trimethoxyflavone (**16**); acacetin (4′-methylapigenin) (**17**); melitric acid A methylester (**18**).

Following fractionations of the butanol (organic-phase C, PIL-C) (Supplementary Materials, Figure S1) and aqueous phases from *S. pilosa* extracts, six compounds were afforded, which were identified by NMR spectroscopy as 3,4-dihydroxyphenylactic acid (**3**), rosmarinic acid (**9**), melitric acid A (**12**), melitric acid A methylester (**18**), clinopodic acid I (**13**) and clinopodic acid O (**15**) (Figure 1). Their NMR spectroscopic data were in accordance with previous reports [4]. A set of NMR spectra and HPLC-PDA-MS chromatograms is available in Supplementary Materials, Figures S3–S10.

**Figure 2:**
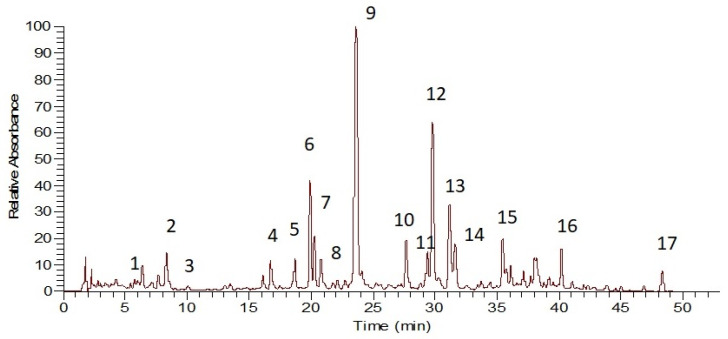
Representative HPLC-PDA-MS chromatogram of the MeOH extract of *Satureja pilosa.* Experimental conditions: column Zorbax SbAq RP-C18 (150 × 3.0 mm), particle size of 3.5 µm (Agilent) at 30 °C. Compounds detected: 12-hydroxyjasmonic acid glucoside (**1**); vicenin 2 (**2**); 3,4-dihydroxyphenyllactic acid (**3**); luteolin 7-*O*-diglucuronide (**4**); luteolin 7-O-rutinoside (**5**); luteolin 7-*O*-glucoside (**6**); luteolin 7-*O*-glucuronide (**7**); hesperidin (hesperitin 7-*O*-rutinoside) (**8**); rosmarinic acid (**9**); luteolin 3′-*O*-glucuronide (**10**); depside tetramer—not identified (**11**); melitric acid A (**12**); clinopodic acid I (**13**); acacetin 7-*O*-rhamnosylglucoside (**14**); clinopodic acid O (**15**); 5,6,3-trihydroxy-7,8,4-trimethoxyflavone (**16**); acacetin (4′-methylapigenin) (**17**).

Although melitric acid A methylester has previously been reported from a closely related Asiatic species, namely *Micromeria biflora* (Buch.-Ham. ex D.Don) Benth., previously known as *Satureja biflora* (Buch.-Ham. ex D.Don) Briq., its presence in *S. pilosa* may be questionable due to the possible reaction of melitric acid A with methanol during the extraction process. HPLC-PDA-MS analyses of the crude extract (Figure 2) and fractions thereof enabled the elucidation of the rest of the constituents.

Their identification (Table 1) was based on co-chromatography with reference standards and lab isolates [25,37], as well as the literature data [38,39,40,41]. The following compounds were identified: 12-hydroxyjasmonic acid glucoside (**1**), vicenin 2 (**2**); luteolin 7-*O*-diglucoside (**4**), luteolin 7-*O*-rutinoside (**5**), luteolin 7-*O*-glucoside (**6**), luteolin 7-*O*-glucuronide (**7**), hesperidin (hesperitin 7-*O*-rutinoside) (**8**), luteolin 3′-*O*-glucuronide (**10**), an unidentified depside tetramer (**11**), acacetin 7-*O*-rhamnosylhexoside (**14**), 5,6,3′-trihydroxy-7,8,4-trimethoxyflavone (**16**) and acacetin (4′-methylapigenin) (**17**). Vicenin 2 (**2**) provided characteristic fragments of neutral losses of 120 amu corresponding to the losses of glucose residues [38]. All luteolin derivatives (**4**–**7**) presented identical UV maxima: a characteristic bathochromic shift of Band I at approximately 346 nm along with the splitting of Band II at 255, 266 nm [42]. Instead, compound **10** was identified as a luteolin 3′-*O*-glucuronide on the basis of its molecular weight and the hypsochromic shift of Band I at 340 nm. In case of a luteolin 4′-*O*-glycosidic derivative, a more pronounced hypsochromic shift would have been observed [43,44]. Furthermore, this constituent has been previously isolated from a closely related Mediterranean member of this genus, namely *S. cuneifolia* Ten. (mentioned with its synonym *S. obovata* Lag. in [39]). UV absorption maxima were also of diagnostic value for the identification of the polymethoxylated flavone **16**. According to its MS fragmentation pattern and molecular weight, the flavone bears 3-hydroxy and 3-methoxy groups. Due to the hypsochromic shift in Band I at 342 nm, a flavone is suggested, whereas the bathochromic shift in Band II at 290 nm is typical of multiple oxygenated substitutions on ring A of the flavonoid. Therefore, the peak could be assigned either to thymonin or to 5,6,3′-trihydroxy-7,8,4′-trimethoxyflavone, both of which are isolated from *S. atropatana* Bonge, a local endemic of northwest Iran [40]. The strong hypsochromic shift of Band I is indicative of a methoxylation on 4′ rather than 3′. Therefore, the peak was assigned to 5,6,3′-trihydroxy-7,8,4′-trimethoxyflavone (**16**). Finally, two more flavonoids **14** and **17** were identified as acacetin 7-*O*-rhamnosylhexoside and acacetin, respectively, based on HPLC-PDA-MS data and previous reports on closely related genera such as *Micromeria* and *Calamintha,* belonging to genus *Satureja sensu lato* [41].

### 2.2. Quantitative Analysis by HPLC-PDA

Quantitation of the major depsides and flavonoids in the examined extracts (crude methanol extract, *n*-butanolic fraction and aqueous phase) showed marked qualitative and quantitative differences (Table 2), as expected due to the fractionation process. The aqueous phase (PIL-D) contained almost exclusively depsides of higher molecular weight, and a smaller quantity of rosmarinic acid, while flavonoids were only found in small amounts. The *n*-butanol phase (PIL-C) contained mainly rosmarinic acid (ca. 6.4% out of a total of 6.71% depsides, *w/w*) and flavonoids of up to 7% (*w/w*), which were mainly represented by luteolin glycosides. The total methanolic extract of *S. pilosa* contained both depsides and flavonoids at a ratio of approximately 2:1.

### 2.3. Influence of Extracts and Pure Compounds on Cell Viability and Apoptosis

**Figure 3:**
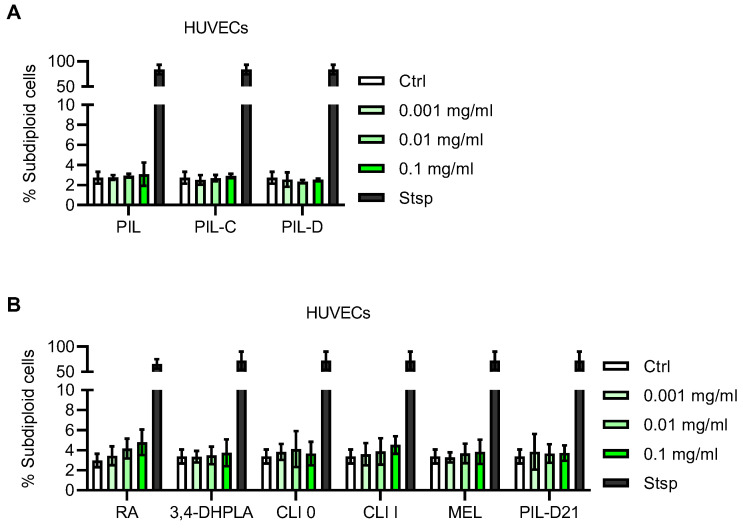
Effects of *Satureja pilosa* extracts and isolated compounds on late-cell apoptosis of HUVECs. Confluent HUVECs were treated with the indicated concentrations of *S. pilosa* extracts (**A**) or isolated compounds and the mixture PIL-D21 (**B**) for 24 h. DMSO (0.1%) served as a vehicle control (ctrl) and staurosporine (1 µM, Stsp) was used as a positive control for the induction of late-cell apoptosis. HUVECs were stained with propidium iodide and late-cell apoptosis was analysed using flow cytometry. PIL, total extract; PIL-C, butanolic fraction; PIL-D, aqueous extract; RA, rosmarinic acid; 3,4-DHPLA, 3,4-dihydroxyphenyllactic acid; CLI O, clinopodic acid O; CLI I, clinopodic acid I; MEL, melitric acid; PIL-D21, mixture of melitric acid and clinopodic acid I. Data are expressed as mean ± SD, *N* = 2.

The cytotoxic effect of *S. pilosa* total (methanolic), *n*-butanol and aqueous extracts (PIL, PIL-C, PIL-D, respectively), as well as of its isolated compounds (rosmarinic acid, 3,4-dihydroxyphenyllactic acid and a mixture 1:1 of clinopodic acid I and melitric acid A), was assessed with the neutral red uptake assay. The results are summarized in Supplementary Materials, Figure S11. Neither the extracts nor the isolated compounds exhibited cytotoxic effects on NIH 3T3 fibroblasts in the concentration range of from 1 × 10^−1^ to 1 × 10^−5^ mg/mL after 24 h, as indicated by the cell viability percentage of above 90%. On the other hand, all the tested substances had toxic effects on NIH 3T3 fibroblasts at the concentration of 1 mg/mL. When evaluated regarding a potential cytotoxic effect in HUVECs using propidium iodide staining, according to Nicoletti et al. [45], none of the *S. pilosa* extracts (PIL, PIL-C, PIL-D) evoked any increase in late-cell apoptosis after 24 h at concentrations up to 1 × 10^−1^ mg/mL (Figure 3A). Similarly, late-cell apoptosis in HUVECs remained unaltered upon treatment for 24 h with isolated compounds (rosmarinic acid (RA), 3,4-dihydroxyphenyllactic acid (3,4-DHPLA), clinopodic acid O (CLI O), clinopodic acid I (CLI I), and melitric acid (MEL), as well as with the mixture of melitric acid and clinopodic acid I (PIL-D21) at concentrations of up to 1 × 10^−1^ mg/mL (Figure 3B). As most of the obtained values showed no toxicity to the extracts in most of the tested concentrations, we selected concentrations between 1 × 10^−1^ and 1 × 10^−5^ mg/mL to assess the wound-healing and anti-inflammatory properties of the focal plant extracts.

### 2.4. Wound-Healing Activity of Extracts and Isolated Compounds on NIH/3T3 Fibroblasts

**Figure 4:**
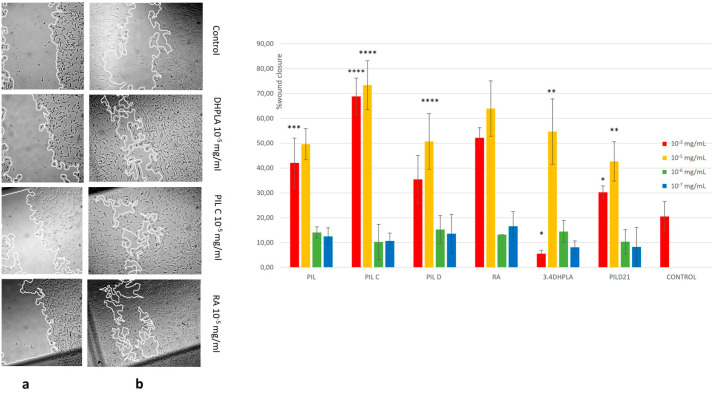
Wound-healing of *Satureja pilosa* extracts and isolated compounds on NIH/3T3 fibroblasts using scratch assay. (**a**) Representative images show the extent of wound closure after 24 h in the untreated (medium) cells, and after treatment with 3,4-DHPLA (10^−5^ mg/mL), PIL-C (10^−5^ mg/mL) and rosmarinic acid (10^−5^ mg/mL). (**b**) Wound-healing activity is expressed as mean of three experiments. Bars ± SD. * *p* < 0.05; ** *p* < 0.01; *** *p* < 0.001; and **** *p* < 0.0001.

The migration activities of the extracts/compounds on 3T3 fibroblasts were evaluated using a scratch assay, which measures the progression of the wound closure on scratch-wounded cells. The total (PIL), the *n*-butanolic (PIL-C) and the aqueous extracts (PIL-D), together with the rosmarinic acid (RA), the 3,4-dihydroxyphenyllactic acid (3,4-DHPLA) and a mixture of melitric and clinopodic acid I (PIL-D21), were examined, focusing on whether they could promote the rate of wound-healing. Thus, 3T3 fibroblasts were treated with different concentrations (between 10^−3^ and 10^−7^ mg/mL) and were observed up to 24 h post scratching. Cells migrated at a higher rate (Figure 4) at concentrations of 10^−5^ mg/mL in all cases (*p* < 0.05), whereas at the even lower concentrations of 10^−6^ and 10^−7^ mg/mL, wound-healing was poor. The *n*-butanolic extract (PIL-C) enhanced cell migration more rapidly at both concentrations and had the highest cell migration rate, with a mean of 68.8% at 10^−3^ mg/mL and 73.4% at 10^−5^ mg/mL. The aqueous extract PIL-D showed a high migration percentage at the concentration of 10^−5^ mg/mL, with 50.6%, as compared to 35.4% at 10^−3^ mg/mL. Among the isolated compounds, RA and 3,4-dihydroxyphenyllactic acid (3,4-DHPLA) reached a migration rate of 64.0% and 54.7%, respectively, at a concentration of 10^−5^ mg/mL but had relatively little effect at the concentration of 10^−3^ mg/mL (52.1% and 5.5%, respectively). Likewise, the mixture of melitric acid and clinopodic acid I (PIL-D21) had a migration rate of 42.6% at 10^−5^ mg/mL and 30.3% at 10^−3^ mg/mL, respectively.

### 2.5. The Majority of Satureja pilosa Extracts and Isolated Compounds Does Not Alter Angiogenic or Inflammatory Activation Processes in Endothelial Cells

**Figure 5:**
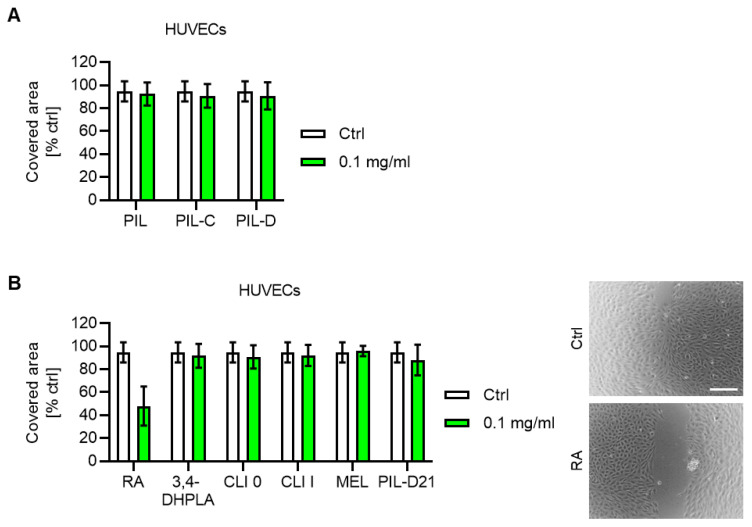
Effects of *Satureja pilosa* extracts and isolated compounds on the undirected migration of HUVECs. A scratch was inflicted on a confluent monolayer of HUVECs. Cells were subsequently treated with 1 × 10^−1^ mg/mL of *S. pilosa* extracts (**A**) or isolated compounds, and the mixture PIL-D21 (**B**). DMSO (0.1%) served as vehicle control (ctrl). HUVECs were allowed to migrate until the untreated control cells had closed the gap (100% covered area). Representative images are shown. Scale bar, 200 µm. PIL, total extract; PIL-C, butanolic fraction; PIL-D, aqueous extract; RA, rosmarinic acid; 3,4-DHPLA, 3,4-dihydroxyphenyllactic acid; CLI O, clinopodic acid O; CLI I, clinopodic acid I; MEL, melitric acid; PIL-D21, mixture of melitric acid and clinopodic acid I. Data are expressed as mean ± SD, *N* = 2.

To explore the potential effect of *S. pilosa* extracts and isolated compounds on a key angiogenic process, i.e., the migration of endothelial cells, a scratch assay was performed. For this, a scratch was inflicted on a monolayer of HUVECs. The cells were subsequently treated with extracts and isolated compounds from *S. pilosa* and were allowed to migrate until the gap in the untreated control cells was closed. At a concentration of 1 × 10^−1^ mg/mL, none of the extracts (PIL, PIL-C, PIL-D) showed any impact on the undirected migration of HUVECs compared to vehicle-treated control cells (Figure 5A). Furthermore, most of the isolated compounds, including 3,4-dihydroxyphenyllactic acid (3,4-DHPLA), clinopodic acid O (CLI O), clinopodic acid I (CLI I) and melitric acid (MEL), as well as the combination of melitric acid and clinopodic acid I (PIL-D21), did not affect the migration of HUVECs (Figure 5B). The caffeic acid ester rosmarinic acid (RA) reduced the migration of HUVECs to approximately 50% compared to the untreated control (Figure 5B).

**Figure 6:**
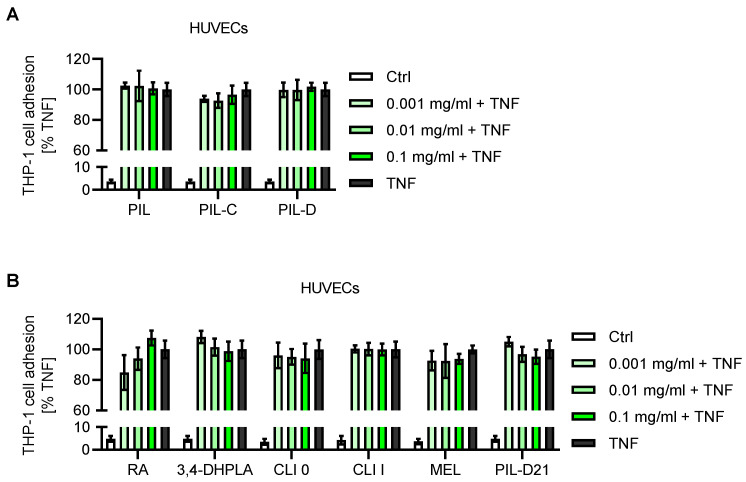
Effects of *Satureja pilosa* extracts and isolated compounds on the leukocyte adhesion to TNF-activated HUVECs. Confluent HUVECs were pretreated for 30 min with the indicated concentrations of *S. pilosa* extracts (**A**) or isolated compounds and the mixture PIL-D21 (**B**), followed by co-treatment with TNF (10 ng/mL) for 24 h. DMSO (0.1%) served as vehicle control (ctrl). Fluorescence-labeled THP-1 cells were allowed to adhere to the monolayer for 5 min, and then non-adherent leukocytes were washed away. Relative cell adhesion was determined by fluorescence measurement. PIL, total extract; PIL-C, *n*-butanolic fraction; PIL-D, aqueous extract; RA, rosmarinic acid; 3,4-DHPLA, 3,4-dihydroxyphenyllactic acid; CLI O, clinopodic acid O; CLI I, clinopodic acid I; MEL, melitric acid; PIL-D21, mixture of melitric acid and clinopodic acid I. Data are expressed as mean ± SD, *N* = 2.

In most pathological skin conditions, both angiogenic and inflammatory processes may contribute to the continuation of the disease. These inflammatory circumstances comprise extensive immune cell activation and expansion, dilation of the dermal microvasculature, and, consequently, accelerated leukocyte recruitment to the injured tissue [46]. To evaluate whether *S. pilosa* influenced inflammatory processes through altering the leukocyte–endothelial cell interaction, a well-established cell-adhesion assay with TNF as the inflammatory stimulus was performed [47,48]. For this, a monolayer of endothelial cells was pretreated with the extracts and isolated compounds from *S. pilosa* for 30 min, and then was inflammatorily activated using TNF, followed by the adhesion of fluorescence-labeled THP-1 cells, a monocytic cell line (Figure 6). It should be noted that only HUVECs were treated in this experimental setup, whereas leukocytes remained untreated to specify the potential actions of the extracts and compounds on the endothelial site. Similar to the results of the scratch assay, leukocyte cell adhesion remained unaffected by either all extracts (PIL, PIL-C, PIL-D), or by isolated compounds such as rosmarinic acid (RA), 3,4-dihydroxyphenyllactic acid (3,4-DHPLA), clinopodic acid O (CLI O), clinopodic acid I (CLI I), and melitric acid (MEL), or by the mixture of clinopodic acid I with melitric acid up to concentrations of 1 × 10^−1^ mg/mL (Figure 6A,B).

### 2.6. Antibacterial Activity Satureja pilosa Extracts and Isolated Compounds

The antibacterial effects of *S. pilosa* extracts (total methanolic, butanolic, aqueous, i.e., PIL, PIL-C, PIL-D), and isolated compounds (rosmarinic acid, 3,4-dihydroxyphenyllactic acid, clinopodic acids O and I, and a mixture 1:1 of clinopodic acid I and melitric acid A) were tested against the Gram-positive bacterium *Staphylococcus aureus* and the Gram-negative bacterium *Pseudomonas aeruginosa*. The results are summarized in Supplementary Materials Table S1. The minimum inhibitory concentration (MIC) of all tested extracts and compounds was found higher than 300 μg/mL. However, higher concentrations were not tested due to the limited quantities of extracts and isolated compounds. Although MIC (e.g., 100% growth inhibition) was not precisely determined, it was observed that bacterial growth was inhibited by from 30% to 50% at 300 μg/mL, depending on the bacterial species.

## 3. Discussion

In the present work, the chemical composition of cultivated *S. pilosa* and the in vitro biological activities of its extracts and isolated compounds were assessed for the first time. A metabolite-specific extraction scheme was applied, focusing on the polar flavonoid and depside content of this range-restricted herb, which was typically extracted in traditional aqueous preparations for medicinal purposes. Overall, the presence of depsides of high molecular weight [4] bearing one or more free carboxylic acids (Figure 1) imposed the use of aqueous methanol to extract them in both ionized and non-ionized forms. The applied chromatographic protocol was based on partition, solid-phase extraction and gel-filtration chromatography (Supplementary Materials, Figures S1 and S2), which enabled the fast removal of terpenoids and sugars, and the acquisition of the sum of depsides. Indeed, the HPLC-PDA-MS analysis of the total fractions revealed the presence of depsides and flavonoid glycosides, thus confirming the above choice. The use of Sephadex LH-20 alternating between polar (aqueous methanol with different ratios of water) and non-polar chromatographic conditions (mixtures of EtOAc, MeOH and water) permitted the faster and more effective isolation of the desired compounds with an upright purity. The targeted chromatographic process, assisted by HPLC-PDA-MS, enabled the selection of the best fractions in every isolation step and permitted the fast isolation of the target compounds such as the high-molecular-weight clinopodic acids. Furthermore, HPLC-PDA-MS was used to characterize the extracts and fractions in a qualitative and quantitative fashion. In total, 18 compounds were identified by NMR and HPLC-PDA-MS. Five depsides were isolated, namely rosmarinic acid (the characteristic metabolite of many Lamiaceae), the trimers melitric acid A and its methylester and, most importantly, the clinopodic acid I (tetramer) and clinopodic acid O (hexamer) of high molecular weight. This is the first report on the phytochemical profile of cultivated *S. pilosa,* and also the first report on clinopodic acids in members of genus *Satureja sensu stricto* [49] or the second report (after *Micromeria biflora*) on clinopodic acids in other members of *Satureja sensu lato* [4].

Following chromatographic and/or HPLC-PDA-MS analysis, representative extracts and selected isolated compounds were subjected to bioactivity assessment, focusing mainly on wound-healing and anti-inflammatory effects. As bacterial infections play a negative role in wound-healing [35,36], the antibacterial activity was also tested. At the same time, a quantitative analysis (Table 2) showed the correlation between the observed activity and the chemical content of the extracts.

At first, the cytotoxic effect of *S. pilosa* extracts and their isolated compounds was assayed on two different cell lines. This is a standard procedure prior to any in vitro bioassay using cell lines. Neither the extracts nor the isolated compounds exerted toxicity in both of the tested cell lines (NIH 3T3 fibroblasts and HUVECs), even at concentrations of up to 1 × 10^−1^ mg/mL. This is important since *S. pilosa* is used for medical, cosmetic and food (melipherous, culinary) purposes.

The wound-healing activity of extracts and pure constituents of *S. pilosa* was tested in NIH/3T3 fibroblasts using the scratch assay. The concentration of 10^−5^ mg/mL exhibited better wound-healing activity. The order of migration activity for extracts was as follows: butanolic (PIL-C 73.4%) > aqueous (PIL-D, 50.6%) > total methanolic (PIL, 49.6%) extracts. Regarding the isolated compounds, the order of mean migration rate was as follows: rosmarinic acid (64.0%) > 3,4-dihydroxyphenyllactic acid (54.7%) > mixture of melitric acid and clinopodic acid I (42.6%). The butanolic fraction (PIL-C) which contained equal amounts of flavonoids and depsides exhibited the best wound-healing activity (73.4%). The aqueous fraction (PIL-D) contained ten-fold the amount of depsides compared to flavonoids and demonstrated a significant wound-healing activity (50.6%). From the above, it seems that flavonoids may promote wound-healing to a greater extent than depsides; however, depsides seem to also significantly enhance the wound-healing activity. Indeed, wound-healing was shown to be promoted in an in vivo study using an enriched flavonoid extract from *Dodonaea viscosa* Jacq. d by upregulating the expression of COL3A, VEGF and bFGF proteins [50]. The weaker activity of the total methanolic fraction compared to the butanolic one might be attributed to the presence of other, probably less active, constituents such as free sugars and chlorophylls.

Although *Satureja* spp. are used to treat several skin ailments, to the best of our knowledge, this is the first report on the wound-healing activity of characterized polar extracts in *Satureja* spp. and isolated constituents thereof. Previous reports on Iranian endemics such as *S*. *khuzistanica* Jamzad and *S*. *rechingeri* Jamzad have demonstrated significant wound-healing activity in a mice model when applied alone or in an alginate hydrogel [51,52]. Nonetheless, as both of the tested extracts in these studies were of an unspecified chemical content, we cannot make any comparison to deduce some useful conclusions. More recently, modern pharmaceutical formulations incorporating *Satureja* spp. extracts and essential oils, such as 3D-printing and nanofibers, have been proposed for diabetic ulcers and antimicrobial wound-dressing, respectively [53,54]. Apart from the essential oil for which a GC-MS analysis is provided by the latter study, there is no other information regarding the extract that is used. Nevertheless, the above reports represent an excellent case of interdisciplinary pharmaceutical science and may show the great potential of range-restricted *Satureja* spp. for applications to treat challenging skin conditions.

Similarly, numerous reports on *Satureja* spp. crude extracts and essential oils suggest anti-inflammatory effects in different in vitro assays (protein denaturation, COX/LOX inhibition, cytokine production, gene expressions of proinflammatory cytokines, etc.) or animal models (carrageenan-induced rat paw edema), but, unfortunately, only in a few cases is the chemical content defined, mostly in terms of essential oils [55,56,57]. In general, most of the studies in the literature focus on essential oils and their main ingredients. Essential oil from the Iranian endemic *S. sahendica* Bornm. is reported to accelerate wound-healing by upregulating the expression of IGF-1, IL-10, FGF-2, VEGF, TGF-ß, and CXCL-1, thus shortening the inflammatory stage and promoting the proliferative phase [58]. In another study, *S. khuzistanica* essential oil dominated by carvacrol has been shown to exert neuroprotective effects in a model of rat brain, in part by modulating NF-κB-regulated inflammation and caspase-3 protein expression [59]. In our experiments, the polar extracts/constituents were selected for study simply because these ingredients are expected to be found in traditionally consumed herbal teas or in aqueous culinary applications. According to our results, excepting rosmarinic acid, which reduced the migration of HUVECs to approx. 50%, none of the *S. pilosa* extracts and isolated compounds altered angiogenic activation. Likewise, both extracts and pure compounds did not affect cell adhesion. Despite the negative results, this is the first report on the effects of *S. pilosa* regarding angiogenic and inflammatory activation processes particularly focusing on clinopodic acids. In general, the literature reports on the biological activities of clinopodic acids are still very scarce, as these are rare constituents among the members of the Lamiaceae family. Most notably, they have been reported to exert strong matrix metalloproteinase-2 and hyaluronidase inhibitory effects, two enzymes related to tissue regeneration, anti-inflammatory, skin-aging, as well as several diseases [60,61]. Undoubtedly, further studies are needed in other experimental models to explore their possible role in skin issues.

Regarding the antibacterial activity, it was not possible to determine the MICs for both compounds and extracts at the tested concentration, which was rather low. Nevertheless, this is the first report on the antibacterial activity of the depsides clinopodic acids I and O, as well as melitric acid A, whereas for rosmarinic acid, the literature reports are contradictory. Several extracts from plants of the Lamiaceae family containing rosmarinic acid, such as *S. hortensis*, are reported to be active [62,63]. On the contrary, rosmarinic acid alone does not show any antibacterial activity against *Staphylococcus aureus* [64]. Similarly, 3,4-dihydroxyphenyllactic acid was found to exert weak antibacterial activity. Quite interestingly, phenyllactic derivatives are reported to exert great antimicrobial potential [65,66]. It seems that the presence of the phenolic hydroxyls may render the molecules more hydrophilic and can impede their interaction with bacterial membranes or other targets inside the bacteria. The same hypothesis might be deduced for the depsides tested in this study, which, besides being polar, are particularly voluminous in order to pass through the bacterial membranes. Like isolated compounds, *S. pilosa* extracts were proved to be rather weak inhibitors of bacterial growth, at least at the tested concentrations. The HPLC-PDA and GC-MS analysis showed the absence of volatile constituents like carvacrol, thymol or linalool, which are well-established as strong antimicrobial components of most of the *Satureja* sp. essential oils [67]. However, these compounds are extant in the analysed essential oil of the cultivated *S. pilosa* (24,); hence, the antimicrobial activity of *S. pilosa* is probably due to the presence of these strong antimicrobial volatiles, without excluding the possible contribution of polar phenolics. Quite recently, and similarly, the flavonoids diosmetin-7-rutinoside and linarin from *S. khuzistanica* were shown to exert synergistic interactions when combined with carvacrol [68]. Therefore, the actual synergism between the phenolics and the volatiles of *S. pilosa* might be an interesting idea to explore in the future. Undoubtedly, the antibacterial activity exerted by *S. pilosa* extracts and purified compounds deserves further investigation.

Considering the study limitations, biological activity assays employing the lipophilic (*n*-hexane) and medium polarity extracts (EtOAc) were not feasible, as their solubility was particularly low in the water-based media.

## 4. Materials and Methods

### 4.1. Plant Sample

The selected biotype of *S. pilosa* was originally collected from Mountain Koula in northern Greece (close to Sminthe village at an altitude of 210 m, 43°13′16″ Ν, 24°52′23″ Ε) using the authorized collection permits 82,336/879 from the 18 May 2019, and 26,895/1527 of 21 April 2021, of the Institute of Plant Breeding and Genetic Resources of the Hellenic Agricultural Organization-Demeter. The voucher and living samples of this selected biotype were taxonomically identified by Dr. Nikos Krigas and maintained in the herbarium and living collections of the Balkan Botanic Garden of Kroussia (BBGK) with the IPEN code (International Plant Exchange Network) GR-1-BBGK-04,2656-06. The living specimens were in vitro propagated and genetically authenticated with DNA barcoding and are currently maintained ex situ in a pilot cultivation on the institute’s grounds ([24], manuscript in preparation).

### 4.2. General Experimental Procedures

^1^H, ^13^C and 2D NMR experiments were recorded at 295 K in CD_3_OD using an Agilent DD2 500 (500.1 MHz for ^1^H-NMR and 125.5 MHz ^13^C-NMR) spectrometer. COSY, HSQC and HMBC were performed using standard Varian microprograms. Column chromatography (CC) was performed on Sephadex LH-20 (MilliporeSigma, Burlington, MA, USA) and Amberlite XAD7HP resin (Supelco, Merck, Darmstadt, Germany) with the solvent mixtures indicated in each case; TLC analyses were carried out using aluminum-coated silica gel plates 60 F_254_ (Merck, Art. 5554). Detection was carried out using UV-light and vanillin/sulfuric acid reagent.

### 4.3. Isolation of the Compounds from S. pilosa

The dried aerial parts of *S. pilosa* (200 g) were finely ground and successively extracted with solvents of increasing polarity, specifically 100% methanol (MeOH, 3× 1 L for 48 h) and MeOH 70% (2× 1 L for 48 h), at room temperature. Apart from maceration and to increase the extraction yield, ultrasonication was additionally employed (30 min maximum at room temperature). The extracts were combined and condensed to dryness. The residue (57.2 g) was redissolved in MeOH 80% and was subjected to liquid–liquid extraction with solvents of increasing polarity, providing four extracts: hexane (organic-phase A, PIL-A, 27.7 g), ethyl acetate (organic-phase B, PIL-B, 8.53 g), *n*-butanol (organic-phase C, PIL-C, 12.5 g) and aqueous extract (8 g). The latter was submitted to Amberlite XAD7HP resin to remove the sugars and enrich the phenolic content (PIL-D, 5.6 g). This extract was subjected to several chromatographic separations over Sephadex LH-20 with MeOH 70%. The obtained fractions were examined by TLC and HPLC-PDA-MS analysis and were recombined to form a total of 9 fractions (PIL-DA to PIL-DI). Fraction PIL-DB (523.3 mg) was subjected to column chromatography (CC) over Sephadex LH-20 with MeOH 80%, and yielded 3,4-dihydroxyphenyllactic acid (**3**) (38.6 mg). Fraction PIL-DE (336.6 mg) was subjected to CC over Sephadex LH-20 with MeOH 80%, and afforded rosmarinic acid (**9**) (80.2 mg). Fraction PIL-DI (793 mg) was subjected to CC over Sephadex LH-20 and eluted successively with a solvent system of increasing polarity (EtOAc:MeOH:H_2_O, 70:30:3 to 30:70:10), and yielded 17 fractions (PIL-DIA to PIL-DIQ). Fraction DIB (eluted with EtOAc:MeOH:H_2_O 70:30:3, 24.9 mg) was subjected to CC over Sephadex LH-20 with MeOH 70%, and afforded melitric acid A methylester (**18**) (4.5 mg). Fraction DIE (eluted with EtOAc:MeOH:H_2_O 70:30:10, 39.9 mg) was subjected to CC over Sephadex LH-20 with MeOH 70%, and afforded melitric acid A (**12**) (7.3 mg). Fraction DIF (eluted with EtOAc:MeOH:H_2_O 70:30:10, 45.3 mg) was subjected to CC over Sephadex LH-20 with MeOH 70%, and afforded a mixture of melitric acid A (**12**) and clinopodic acid I (**13**) (10.5 mg), as well as clinopodic acid I (**13**) (5.2 mg). Fraction DIM (eluted with EtOAc:MeOH:H_2_O 60:40:10, 227.3 mg) was subjected to CC over Sephadex LH-20 with MeOH 70%, and afforded clinopodic acid O (**15**) (25.9 mg).

### 4.4. Chemicals and Standards

The solvents used for the isolation of the flavonoids were of reagent grade, whereas the solvents used for HPLC analysis were HPLC grade. All solvents were purchased from Sigma-Aldrich (MilliporeSigma, Burlington, MA, USA). Water was purified by a Milli-Qplus system from Millipore (Milford, MA, USA). Sephadex LH-20 was purchased from Sigma-Aldrich. Nylon filters (0.45 µm pore size) were from Agilent (Agilent Technologies, Palo Alto, CA, USA). Rosmarinic acid (97% purity) was purchased from Alfaesar (Kandel, Germany). Apigenin 7-*O*-glucoside (≥99% purity) and luteolin 7-*O*-glucoside (>98% purity) were purchased from Extrasynthèse (Genay, France). A series of stock solutions were prepared and kept at −20 °C in 100% methanol. From these stock solutions, a series of fresh working solutions were prepared immediately prior to analysis.

### 4.5. HPLC-PDA-MS Analysis Instrumentation

Analysis was carried out using an HPLC-PDA-MS Thermo Finnigan system (LC Pump Plus, Autosampler, Surveyor PDA Plus Detector) interfaced with an ESI MSQ Plus (Thermo Finnigan, San Jose, CA, USA) and equipped with an Xcalibur 2.1 software. The same column, timetable and flow rate were used during the HPLC-MS analyses. The mass spectrometer operated in both negative and positive ionization modes, scan spectra ranged from *m*/*z* 100 to 1200, gas temperature was 350 °C, nitrogen flow rate was 10 L/min, and capillary voltage was 3000 V. The cone voltage was in the range 60–120 V. The column was a SB-Aq (Agilent, Santa Clara, CA, USA) RP-C18 column (150 mm × 3 mm) with a particle size of 3.5 µm maintained at 30 °C. The eluents were H_2_O at pH 2.8 by formic acid (0.05% *v*/*v*) (A) and acetonitrile (B), with a flow rate of 0.4 mL/min. The samples were analyzed using a gradient program, as follows: 0–5 min, 85%A; 5–15 min 85–78%A; 15–20 min 78%A; 20–22 min 78–75%A; 22–27 min 75%A; 27–37 min 75–60%A; 37–44 min 60%A; 44–48 min, 60–85%A; 48–53 min, 85%A. A total of 5 μL of solution was injected into the samples. The UV–vis spectra were recorded between 220 and 600 nm and the chromatographic profiles were registered at 288, 330 and 350 nm.

### 4.6. Qualitative and Quantitative Determination of Phenolic Compounds

Identification of the constituents of *S. pilosa* was performed by examination of their retention time, UV, and MS data, as well as by comparison with authentic reference samples and the literature data [38,39,40,41]. These results are shown in Table 1 and Table 2. The identification of compounds **2**, **8**, **11**, **12**, **14** and **17** was carried out by 1D and 2D NMR and by HPLC-PDA-MS. The identification of the rest of the constituents was performed by HPLC-PDA-MS. For the quantitative determination, the method of external standard was applied. The linearity range of responses of the standards was determined at five concentration levels, with two injections for each level. Calibration graphs for HPLC were recorded with amounts ranging from 0.02 to 0.06 μg of the standard solution for luteolin 7-*O*-glucoside; for apigenin 7-*O*-glucoside, the amounts ranged from to 0.12 to 0.36 μg; and for rosmarinic acid, the results ranged from 0.02 tο 0.07 μg. Measurements were performed at 330 nm for rosmarinic acid, relative depsides and apigenin derivatives, and at 350 nm for luteolin derivatives. Results were adjusted using a molecular-weight correction factor.

### 4.7. Biological Activities of Satureja pilosa Extracts and Constituents

#### 4.7.1. Equipment and Reagents

The cells were cultivated in a InCO2 Memmert incubator (Schwabach, Germany); the vertical flow bench was a Telstar abductor PV100 (Barcelona, Spain). An Axiovert 25 ZEISS (Oberkochen, Germany) inverted microscope and a Fluostar Galaxy BMG Microplate Photometer (Ortenberg, Germany) were used. Centrifuge was a Hettich Roto Sienta III (Tuttlingen, Germany). The laboratory drying oven was from Memmert and the liquid-nitrogen freezing cells’ container was a 34XT Taylor Wharton (Cambridge Scientific, Merck KGaA, USA). Single-channel and multi-channel micro-pipettes were from Nichiryo (Tokyo, Japan). Tissue culture flasks of 25 cm^2^ and 75 cm^2^, flat-bottomed 96-well tissue and 24-well culture plates, and centrifuge tubes of 50 mL were all from SPL Lifesciences (Pocheon-si, Korea). A DSLR camera, PowerShot G5 X, (zoom lens 7.2–28.8 mm 1:2.0–3.0) (Canon, USA) was used for the imaging of the cells. ImageJ software [69] and Adobe Photoshop were used for the editing.

#### 4.7.2. Chemicals and Biochemicals

Dimethyl sulfoxide (DMSO), absolute ethanol, glacial acetic acid, propidium iodide, staurosporine and triton X-100 were of analytical grade and were purchased from Sigma-Aldrich (Merck KGaA, USA). Neutral red dye was from Apollo Scientific Sigma (Bredbury, Stockport, Cheshire, UK). Sodium citrate was obtained from Carl Roth (Karlsruhe, Germany); collagenase A was from Roche (Basel, Switzerland). Τhe Collagen G used for pre-coating the plastic ware on which HUVECs were grown was from Biochrom (Berlin, Germany). Recombinant human TNFα was purchased from PeproTech (Rocky Hill, NJ, USA) and the fluorescent dye Green CMFDA was obtained from Cayman Chemical (Ann Arbour, MI, USA).

#### 4.7.3. Cell Lines

NIH/3T3, a fibroblast cell line that was isolated from a mouse NIH/Swiss embryo, was kindly provided by Dr D. Kletsas, National Center for Scientific Research Demokritos (Greece). NIH/3T3 cells were grown in Dulbecco’s Modified Eagles Medium (DMEM) supplemented with 10% fetal bovine serum (FBS; PAN-Biotec, Germany), 1% antibiotic-antimycotic solution (Biosera, France) and 4 mM L-glutamine, and were subcultured after reaching 80–90% confluency using trypsin/EDTA (Biosera).

Human umbilical vein endothelial cells (HUVECs) were isolated as previously described [70] using a special permission (reference number W1/21Fü), granted for the use of anonymized human material on 15 September 2021, issued by the head of the Research Ethics Committee/Institutional Review Board of the Goethe University in Frankfurt Germany. In brief, the veins from human umbilical cords were filled with collagenase A (0.1 g/L; Roche, Basel, Switzerland) and incubated for 45 min to detach the endothelial cells. Endothelial cells were seeded to pre-coated (collagen G, 10 µg/mL; Biochrom) plastic products and cultivated in an endothelial cell growth medium (ECGM; PELOBiotech, Planegg/Martinsried, Germany) containing 10% FBS (Biochrom, Berlin, Germany), 100 U/mL penicillin, 100 µg/mL streptomycin (PAN-Biotech, Aidenbach, Germany), 2.5 µg/mL amphotericin B (PAN-Biotech) and a supplement mixture (PELOBiotech). HUVECs were split in a 1:3 ratio. For experimental purposes, HUVECs were only used in passage 3.

The human monocytic cell line THP-1 was purchased from the German collection of Microorganisms and Cell Cultures (DSMZ, Braunschweig, Germany) and was cultivated in Royal Park Memorial Institute 1640 (RPMI; PAN-Biotech), supplemented with 10% FCS (Biochrom), 100 U/mL penicillin and 100 µg/mL streptomycin (PAN-Biotech). THP-1 cells were used for experimental purposes up to passage 30. Cultivation of all cell types was conducted with constant humidity in an atmosphere of 5% CO_2_ at 37 °C.

For the antibacterial activity, the methicillin-resistant *Staphylococcus aureus* 1552 and the carbapenem-resistant *Pseudomonas aeruginosa* 1773 were used. These clinical strains were identified and characterized by standard methods (kindly provided by Prof. S. Pournaras, School of Medicine, National and Kapodistrian University of Athens). Bacteria were routinely grown in Mueller–Hinton Bboth (Lab M, UK) or Mueller–Hinton agar (Lab M, UK) at 37 °C.

#### 4.7.4. Cytotoxicity Experiments

##### Cell Viability of NIH/3T3 Fibroblasts

NIH/3T3 cells were plated in 96-well flat-bottomed microplates at a density of 10,000 cells/well in serum-containing medium. After 24 h, to ensure cell attachment, the medium was changed to a reduced serum (5% FBS) DMEM containing six serial dilutions of the test extracts (1–1 × 10^−5^ mg/mL). Cultures incubated with the corresponding vehicle concentrations (DMEM, 5% FBS) served as negative controls, whereas cultures containing a standard substance with known cytotoxicity (sodium lauryl sulfate (SLS)) acted as a positive control. Following a 24 h incubation, the medium was replaced with neutral red dye (dissolved at a final concentration of 0.05 mg/mL in serum-free DMEM) and incubated for further 3 h at 37 °C to allow for the uptake of the vital pigment into the lysosomes of viable uninjured cells. Then, the dye was extracted, and the uptake was quantified. Absorbances were recorded at 570 nm and, from the absorbance values, cell viability was calculated as follows:

$$\mathrm{Cell}\;\mathrm{viability}\;\left(\%\right)=\frac{\mathrm{mean}\;\mathrm{absorbance}\;\mathrm{of}\;\mathrm{test}\;\mathrm{sample}}{\mathrm{mean}\;\mathrm{absorbance}\;\mathrm{of}\;\mathrm{negative}\;\mathrm{control}\;}\times 100$$

##### Late Apoptosis of HUVECs

To assess the potential effects of *S. pilosa* extracts on the late-cell apoptosis of HUVECs, propidium iodide (PI) staining was conducted according to Nicoletti et al. [45]. In brief, confluent HUVECs were treated for 24 h with the extracts, as indicated in the respective figure legends. Treatment with 1 µM staurosporine (Stsp) for 24 h was used as a positive control to evoke late-cell apoptosis. After the end of the treatment period, cell culture supernatants, which contain detached apoptotic cells, were collected and the remaining cells were detached using trypsin/EDTA (Biochrom, Berlin, Germany). The supernatant and detached cells were combined and incubated with a hypotonic staining solution (50 µg/mL PI), 0.1% Triton X-100 and 0.1% sodium citrate. Median values of 10,000 events per sample were recorded to evaluate the percentage of cells with subdiploidic DNA content using a FACSVerse flow cytometer (BD Biosciences, San Jose, CA, USA).

#### 4.7.5. Wound-Healing Experiments

##### Wound-Healing Assay in NIH/3T3 Fibroblasts

The stimulating effect of all samples on the migration of NIH/3T3 fibroblasts was determined as described by Fronza et al. [71], with some modifications. The cells were seeded into 24-well tissue culture dishes at a concentration of 5 × 10^4^ cells/mL and cultured in medium containing 10% FBS to nearly confluent cell monolayers. Once the monolayer was yielded, the culture medium was replaced by PBS and a linear wound was generated using a sterile 200 μL pipette tip. PBS and any cellular debris were removed and DMEM medium with FBS (0.2%) was added (control group). DMEM medium with FBS (15%) was used as a positive control. The extracts and the isolated compounds of *S. pilosa* were added at different concentrations (from 10^−3^ mg/mL to 10^−7^ mg/mL) in starvation medium (DMEM containing 0.2% FBS) and incubated for 24 h at 37 °C under 5% CO_2_. Experiments were conducted in triplicate. The cell’s ability to proliferate was inhibited by adding 0.2% FBS to assure that wound closure was only due to cell migration. Cell migration was observed under an inverted microscope with phase contrast, with 4× objective magnification, which was found to be the most suitable for observing the entire width of the wound. Images of the incision closure were acquired at the onset (time = 0) and after 24 h. The images were analyzed using Image J software for Windows. The area of the gap was calculated at the onset and at 24 h, using the following formula:

$$\mathrm{wound}\;\mathrm{closure}\;\%\;=\frac{\left(A-B\right)*100}{A}$$

where:

A = wound area (t = 0);
B = wound area (t = 24 h).

##### Undirected Migration of HUVECs

The undirected migration of endothelial cells was examined using a scratch assay. For this, a scratch was inflicted on a confluent monolayer of HUVECs using a pipette tip, followed by the addition of fresh cell growth medium. Subsequently, compound treatment was conducted, as indicated in the respective figure legend, and HUVECs were allowed to migrate for 10 h until the scratch from the control treatment (0.01% DMSO) was closed again (representing 100% migration). Cells treated with vehicle control (0.01% DMSO) in starvation medium served as a negative control (equalling 0% migration). Scratches were observed using a Leica DM IL LED inverted microscope (Leica Microsystems, Wetzlar, Germany), and relative cell migration in relation to the positive and negative controls was analyzed using ImageJ (software version 1.49k).

#### 4.7.6. Leukocyte Cell Adhesion Assay

The leukocyte–endothelial cell interaction was investigated using a leukocyte cell adhesion assay. In brief, confluent HUVECs were pretreated with *S. pilosa* extracts for 30 min followed by the inflammatory activation of HUVECs by TNF (10 ng/mL; PeproTech, Rocky Hill, NJ, USA) for 24 h. THP-1 cells were fluorescence-labeled using Green CMFDA (Cayman Chemical, Ann Arbour, MI, USA). Then, 1.5 × 10^5^ THP-1 cells per well were allowed to adhere to the HUVEC monolayer for 5 min. Non-adherent THP-1 cells were washed away and the relative amount of adhered THP-1 cells was determined by fluorescence measurement (ex: 485 nm; em: 535 nm) using a microplate reader (SPECTRAFluor Plus, Tecan, Männedorf, Switzerland).

#### 4.7.7. Statistical Analysis

GraphPad Prism 8 software (GraphPad Software, San Diego, CA, USA) was used for statistical analysis for the experiments conducted with NIH/3T3 fibroplasts. The experiments were carried out in triplicate. The Shapiro–Wilk test was performed for the normality test. As the data followed a normal distribution, statistical significance was tested with one-way Anova and the differences were considered statistically significant at *p* < 0.05. Experiments performed with HUVECs were conducted with two individual donors (*n* = 2). All other results were obtained from at least three replicates and expressed as mean ± standard deviation. Statistical significance between groups was determined by one-way ANOVA followed by Tukey’s test for post hoc comparison. Mean values were considered statistically different when *p* < 0.05.

#### 4.7.8. Determination of Minimum Inhibitory Concentration (MIC)

The antibacterial activity was tested against methicillin-resistant *Staphylococcus aureus* 1552 clinical strain and carbapenem-resistant *Pseudomonas aeruginosa* 1773 clinical strain. The bacteria were routinely grown in Mueller–Hinton broth (Lab M, Bury, UK) or Mueller–Hinton agar (Lab M) at 37 °C [72]. The determination of MICs was carried out using sterile 96-well polystyrene microtiter plates (Kisker, Germany) according to Tsavea and Mossialos [73], with some minor modifications. In brief, overnight bacterial cultures cultured in Mueller–Hinton broth were adjusted to 0.5 McFarland turbidity standard (approx. 1.5 × 10^8^ CFU/mL). Approximately 5 × 10^4^ CFUs in 10 μL Mueller–Hinton broth were added to 190 μL Mueller–Hinton broth containing the tested compound at 50, 100, 150, 200, 250 and 300 μg/mL. Each compound was tested in triplicate. Control wells contained Mueller–Hinton broth inoculated with bacteria. Optical density (OD) was determined at 630 nm using an ELx808 Absorbance Microplate Reader (BioTek, Vermont, USA) just prior to incubation (t = 0) and after 24 h incubation (t = 24) at 37 °C. The OD for each replicate well at t = 0 was subtracted from the OD of the same replicate well at t = 24. The growth inhibition at each compound concentration was determined using the following formula: Percentage (%) of inhibition = 1 − (OD test well/OD of corresponding control well) × 100. The minimum inhibitory concentration was determined as the lowest compound concentration that resulted in 100% growth inhibition.

## 5. Conclusions

In the present investigation, *S. pilosa* from a pilot sustainable cultivation was chemically characterized for the first time and further evaluated regarding its in vitro biological effects related to skin conditions. The phytochemical analyses by HPLC-PDA-MS and NMR resulted in 18 compounds and revealed that *S. pilosa* is a rich source of phenolic compounds, especially depsides. Among the identified compounds, clinopodic acids were characterized for the first time in members of *Satureja sensu stricto* or for the second time in members of the genus *Satureja sensu lato*; thus, these acids are still rare and intriguing. The examined extracts and the isolated components were not found to be toxic in two different cell lines (3T3 fibroblasts and HUVEC cells), underlying the lack of toxicity of this medicinal–aromatic herb in traditional topical or oral applications over a short period of time. The examined butanolic extract and rosmarinic acid showed a significant wound-healing effect. Both flavonoids and depsides seemed to play a major role in wound-healing activity. The anti-inflammatory and antibacterial activity was not significant, but further studies are needed to explore possible interactions and/or synergistic effects between the volatile and the non-volatile fractions of this interesting range-restricted plant species.

## Figures and Tables

**Table 1:** MS fragmentation and UV-vis absorption data of the compounds detected in the MeOH extract of *Satureja pilosa*.

Νο.	Rt (min)	UV (nm)	*m*/*z* (−) Negative Mode	*m*/*z* (+) Positive Mode	Identification	Mode of Identification
**1**	5.7	233	207 [M − Glc-H]^−^, 387 [M − H]^−^	227 [A + H]^+^	12-hydroxyjasmonic acid glucoside	UV/MS, lab isolate [37]
**2**	8.4	271, 334	353 [M-2 x Glc residue − H]^−^, 473 [M-Glc residue − H]^−^, 592.8 [M − H]^−^	355 [M-2 x Glc residue- + H]^+^, 595 [M + H]^+^	vicenin 2 (apigenin 6,8-di-*C*-glucoside)	UV/MS, lab isolate [37]
**3**	10.1	281	197 [M − H]^−^	181 [M-H_2_O + H]^+^	3,4-dihydroxyphenyllactic acid	NMR, UV/MS
**4**	16.7	254, 266sh, 347	285 [A − H]^−^, 609 [M − H]^−^	287 [A + H]^−^, 611 [M + H]^+^	luteolin 7-*O*-dihexoside	UV/MS
**5**	18.8	255, 266sh, 346	285 [A − H]^−^, 593 [M − H]^−^	287 [A + H]^−^, 449 [M-Rha group + H]^+^, 595 [M + H]^+^	luteolin 7-*O*-rutinoside	UV/MS, lab isolate [25]
**6**	19.9	255, 266sh, 346	285 [A − H]^−^, 447 [M − H]^−^	287.0 [A + H]^−^, 449 [M + H]^+^	luteolin 7-*O*-glucoside	UV/MS, std
**7**	20.3	253, 266sh, 346	285 [A − H]^−^, 461 [M − H]^−^	287 [A + H]^−^, 463 [M + H]^+^	luteolin 7-*O*-glucuronide	UV/MS, lab isolate [37]
**8**	20.8	284, 328sh	301 [A − H]^−^, 609 [M − H]^−^	303 [A + H]^−^, 611 [M + H]^+^, 633 [M + Na]^+^	hesperidin (hesperitin 7-*O*-rutinoside)	UV/MS, lab isolate [25]
**9**	23.6	288, 328	161, 197, 359 [Μ − H]^−^	163, 361 [M + H]^+^	rosmarinic acid	NMR, UV/MS
**10**	27.7	268, 340	285 [A − H]^−^, 461 [M − H]^−^	287 [A + H]^−^, 463 [M + H]^+^	luteolin 3′-*O*-glucuronide	UV/MS, [39]
**11**	29.4	287, 329	357, 519, 717 [M − H]^−^	741 [M + Na]^+^	depside tetramer—not identified	UV/MS
**12**	29.8	299, 323	135, 161, 179 [caffeic acid − H]^−^, 197 [DPLA − H]^−^, 295 [M-caffeic acid-H_2_O-CO_2_ − H]^−^, 359 [M-caffeoyl group − H]^−^, 493 [M-CO_2_ − H]^−^, 537 [M − H]^−^	163, 297, 341 [M-DPLA + H]^+^, 521 [M-H_2_O + H]^+^, 561 [M + Na]^+^	melitric acid A	NMR, UV/MS
**13**	31.1	287, 334	295 [M-caffeic acid-DPLA-CO_2_ − H]^+^, 321 [M-caffeic acid-DPLA-H_2_O − H]^−^, 339 [M-caffeic acid-DPLA − H]^−^, 519 [M-DPLA − H]^−^, 717 [M − H]^−^	181 [caffeic acid + H]^+^, 341 [M-caffeic acid-DPLA + H]^+^, 521 [M-DPLA + H]^+^, 741 [M + Na]^+^	clinopodic acid I	NMR, UV/MS
**14**	31.58	267, 332	283 [A − H]^−^, 591 [M − H]^−^ 637 [M + HCOO]^−^	285 [A + H]^−^, 447, 593 [M + H]^+^	acacetin 7-*O*-rutinoside	UV/MS, [41]
**15**	35.45	267, 335	339, 519 [M-DPLA-358 − H]^−^, 665, 1075 [M − H]^−^	323, 359 [M-2x DPLA-358 + H]^+^ [M-2x DPLA + H]^+^, 681 [M-2x DPLA + H]^+^, 879 [M-DPLA + H]^+^, 1099 [M + Na]^+^	clinopodic acid O	NMR, UV/MS
**16**	40.2	290, 342	269 [A − H]^−^, 329 [M-2 methyl groups − H]+, 344 [M-methyl group − H]^−^, 359 [M − H]^−^	271 [A + H]^+^, 332 [M-2 methyl groups + H]^+^, 346 [M-methyl group + H]^+^, 361 [M + H]^+^	5,6,3′-trihydroxy-7,8,4′-trimethoxyflavone	UV/MS, [40]
**17**	48.30	268, 329	268 [M-methyl group − H]^−^, 283 [M − H]^−^	242 [M-methyl group-CO_2_ + H]^+^, 270 [M-methyl group + H]^+^, 285 [M + H]^+^	acacetin (4′-methylapigenin)	UV/MS, [41]

A: aglycon; DPLA: 3,4-dihydroxyphenyllactic acid; Glc: glucose; Rha: rhamnose.

**Table 2:** Amounts of flavonoids and depsides in the polar extracts of *Satureja pilosa* (*n* = 3). Results expressed as percentage (%) *w/w*.

Compound Name	Total MeOH—PIL	PIL-C (*n*-BuOH Fraction)	PIL-D (Aqueous Fraction)
luteolin 7-*O*-dihexoside	1.02 ± 0.01	1.37 ± 0.01	1.72 ± 0.01
luteolin 7-*O*-rutinoside	1.00 ± 0.01	1.35 ± 0.01	-
luteolin 7-*O*-glucoside	0.83 ± 0.01	1.12 ± 0.01	-
luteolin 7-*O*-glucuronide	0.81 ± 0.01	1.10 ± 0.01	1.34 ± 0.04
luteolin 3′-*O*-glucuronide	0.80 ± 0.01	1.14 ± 0.02	-
acacetin 7-*O*-rutinoside	0.53 ± 0.10	0.98 ± 0.14	-
**Total flavonoids**	**4.99 ± 0.08**	**7.07 ± 0.11**	**3.06 ± 0.04**
rosmarinic acid	3.51 ± 0.10	6.38 ± 0.10	4.04 ± 0.13
cllinopodic acid I	1.00 ± 0.07	0.26 ± 0.01	4.87 ± 0.06
cllinopodic acid O	0.91 ± 0.10	0.06 ± 0.01	8.71 ± 0.11
melitric acid A	2.28 ± 0.05	-	10.66 ± 0.18
unknown depside	0.53 ± 0.03	-	2.47 ± 0.11
**Total depsides**	**8.24 ± 0.27**	**6.71 ± 0.10**	**30.75 ± 0.31**

## Data Availability

NMR data is contained within the Supplementary Materials, HPLC-PDA-MS data presented in this study are available on request from the corresponding author.

## Supplementary Materials

The following supporting information can be downloaded at: https://www.mdpi.com/article/10.3390/plants12244114/s1, Figure S1: Scheme of the whole extraction protocol, analysis and biological assays; Figure S2: Scheme of the isolation process; Figure S3: Representative HPLC-PDA-MS chromatogram of the Butanol extract (organic-phase C) of *Satureja pilosa*; Figure S4: Representative HPLC-PDA-MS chromatogram of the aqueous phase of *Satureja pilosa*; Figure S5: 1H-NMR spectrum (CD_3_OD, 500 ΜHz) of 3,4-dihydroxyphenylactic acid; Figure S6: ^1^H-NMR spectrum (CD_3_OD, 500 ΜHz) of rosmarinic acid; Figure S7: ^1^H-NMR spectrum (CD_3_OD, 500 ΜHz) of melitric acid A; Figure S8: ^1^H-NMR spectrum (CD_3_OD, 500 ΜHz) of melitric acid A methylester; Figure S9: 1H-NMR spectrum (CD_3_OD, 500 ΜHz) of clinopodic acid I; Figure S10: ^1^H-NMR spectrum (CD_3_OD, 500 ΜHz) of clinopodic acid O; Figure S11: The effects of different extracts of *Satureja pilosa*, rosmarinic acid, 3,4-dihydrophenyllactic acid and the mixture of clinopodic acid I/melitric acid A on the cell viability of 3T3 fibroblasts; Table S1: Antibacterial activity of representative compounds from *Satureja pilosa* against *Staphylococcus aureus* and *Pseudomonas aeruginosa,* expressed as MIC values.
