# Supplementary material for: HPLC- and NMR-Based Chemical Profiling, Wound-Healing Potential, Anti-Inflammatory and Antibacterial Activities of Satureja pilosa (Lamiaceae), a Neglected Medicinal–Aromatic Herb
Source: Plants (Basel). 2023 Dec 8;12(24):4114. doi: 10.3390/plants12244114 (PMC10747026; doi:10.3390/plants12244114)
Supplement: Supplementary file 1 [file plants-12-04114-s001.zip › plants-2667427-supplementary.pdf]

HPLC- and NMR-based chemical profiling, wound healing potential, anti-inflammatory and antibacterial activities of *Satureja pilosa* (Lamiaceae), a neglected medicinal-aromatic herb

Christina Panagiotidou, Luisa D. Burgers, Chara Almpani, Christina Tsadila, Nikos Krigas, Dimitris Mossialos, Michail Christou Rallis, Robert Fürst[f], Anastasia Karioti,*

Supplementary materials

**Figure S1**. Scheme of the whole extraction protocol, analysis and biological assays	**2**
**Figure S2**. Scheme of the isolation process	**3**
**Figure S3.** Representative HPLC-PDA-MS chromatogram of the Butanol extract (Organic phase C) of *Satureja pilosa.*	**4**
**Figure S4.** Representative HPLC-PDA-MS chromatogram of the aqueous phase of *Satureja pilosa*	**5**
**Figure S5.** ^{1}H-NMR spectrum (CD_3OD, 500 MHz) of 3,4-dihydroxyphenylactic acid	**6**
**Figure S6.** ^{1}H-NMR spectrum (CD_3OD, 500 MHz) of rosmarinic acid	**6**
**Figure S7.** ^{1}H-NMR spectrum (CD_3OD, 500 MHz) of melitric acid A	**7**
**Figure S8.** ^{1}H-NMR spectrum (CD_3OD, 500 MHz) of melitric acid A methylester	**8**
**Figure S9.** ^{1}H-NMR spectrum (CD_3OD, 500 MHz) of clinopodic acid I	**9**
**Figure S10.** ^{1}H-NMR spectrum (CD_3OD, 500 MHz) of clinopodic acid O	**10**
**Figure S11**. The effects of different extracts of *S. pilosa*, rosmarinic acid, 3,4-dihydrophenyllactic acid and the mixture of clinopodic acid I/melitric acid A on cell viability of 3T3 fibroblasts	**11**
**Table S1**. Antibacterial activity of representative compounds from *Satureja pilosa* *Staphylococcus aureus* and *Pseudomonas aeruginosa* expressed as MIC value	**11**

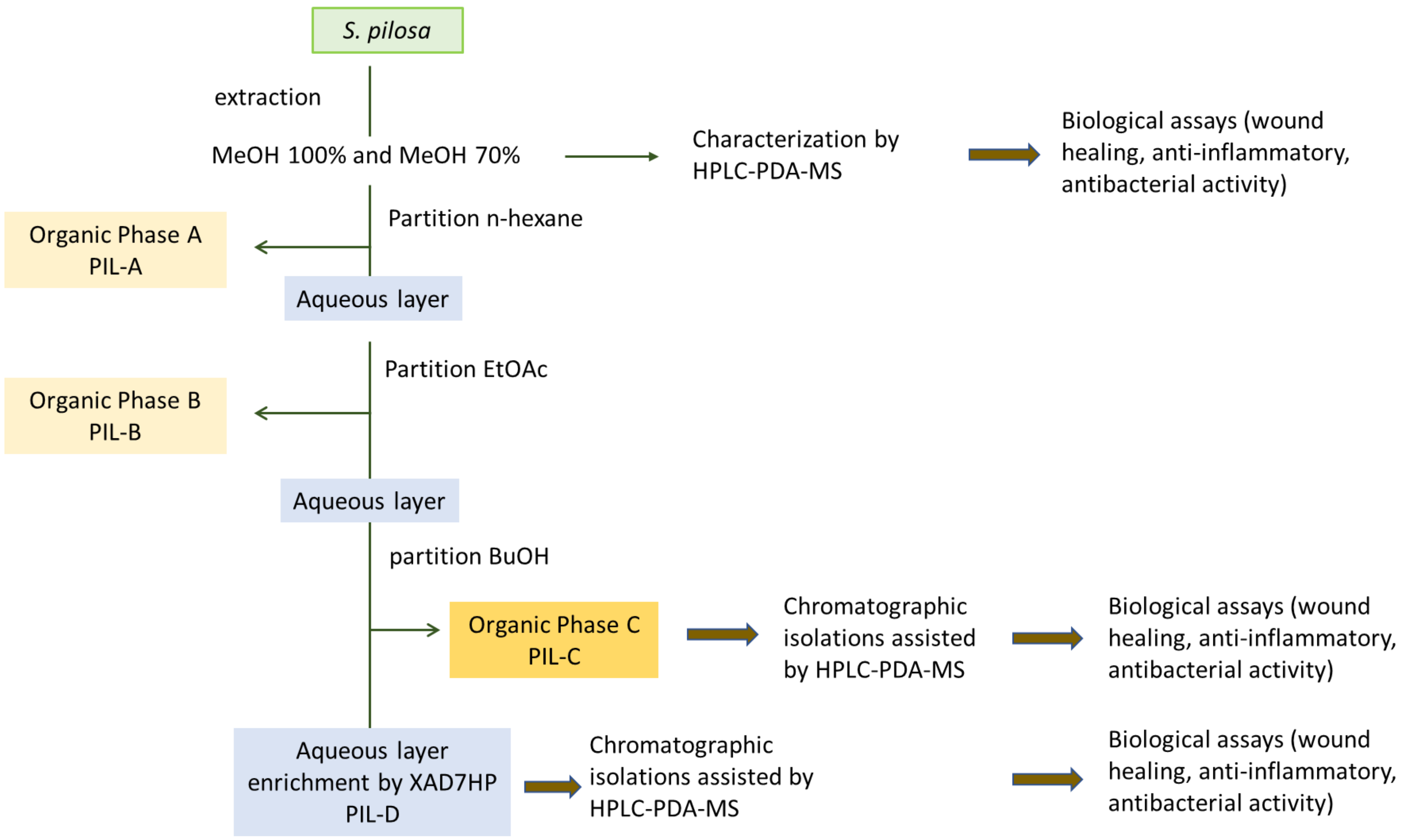

Figure S1. Scheme of the whole extraction protocol, analysis and biological assays for cultivated *Satureja pilosa*.

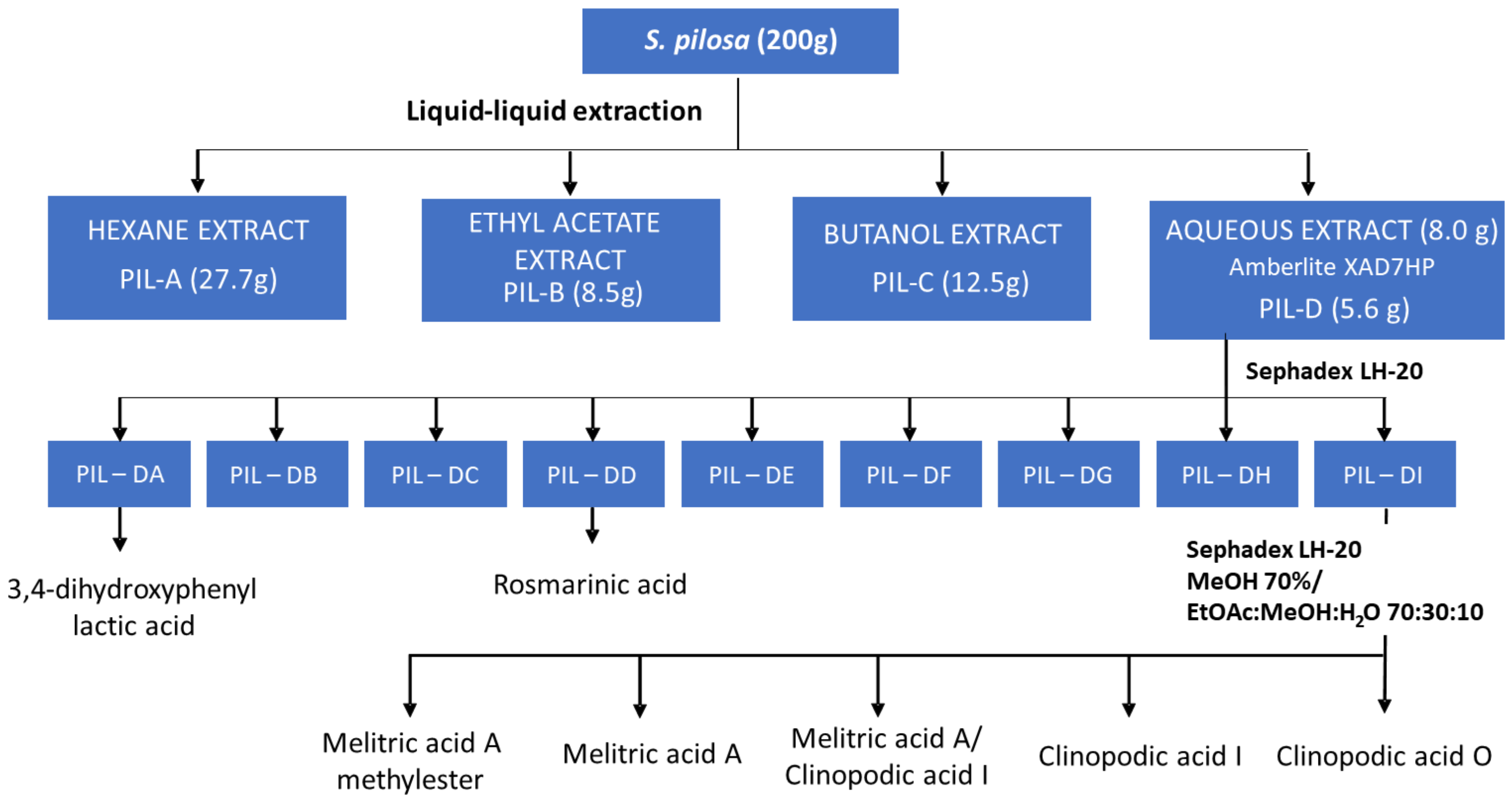

Figure S2. Scheme of the isolation process.

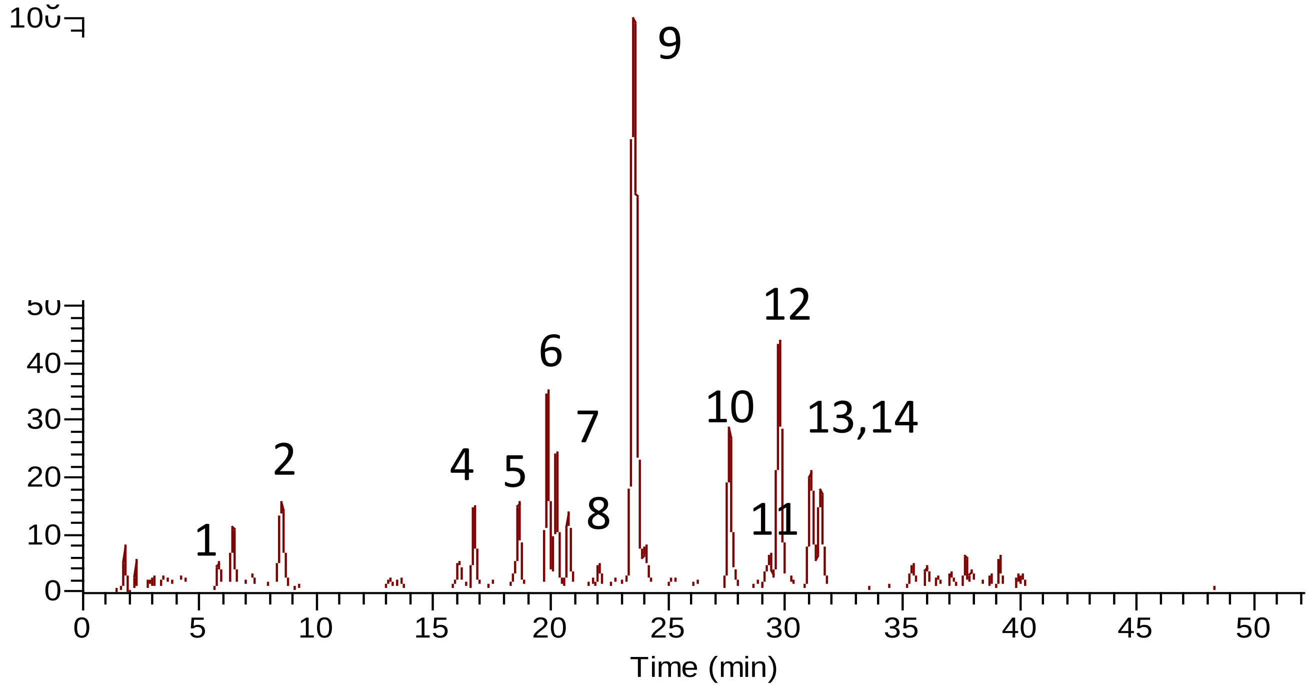

Figure S3. Representative HPLC-PDA-MS chromatogram of the Butanol extract (Organic phase C) of *Satureja pilosa*. Experimental conditions: column: Zorbax SBAq RP-C18 (150 x 3.0 mm), particle size of 3.5µm (Agilent) at 30 °C. Compounds detected: 12-hydroxyjasmonic acid glucoside (**1**); vicenin 2 (**2**); luteolin-7-*O*-diglucuronide (**4**); luteolin-7-*O*-rutinoside (**5**); luteolin-7-*O*-glucoside (**6**); luteolin-7-*O*-glucuronide (**7**); hesperidin (hesperitin-7-*O*-rutinoside) (**8**); rosmarinic acid (**9**); luteolin-3′-*O*-glucuronide (**10**); depside tetramer – not identified (**11**); melitric acid A (**12**); clinopodic acid I (**13**); acacetin-7-*O*-rhamnosyl-glucoside (**14**).

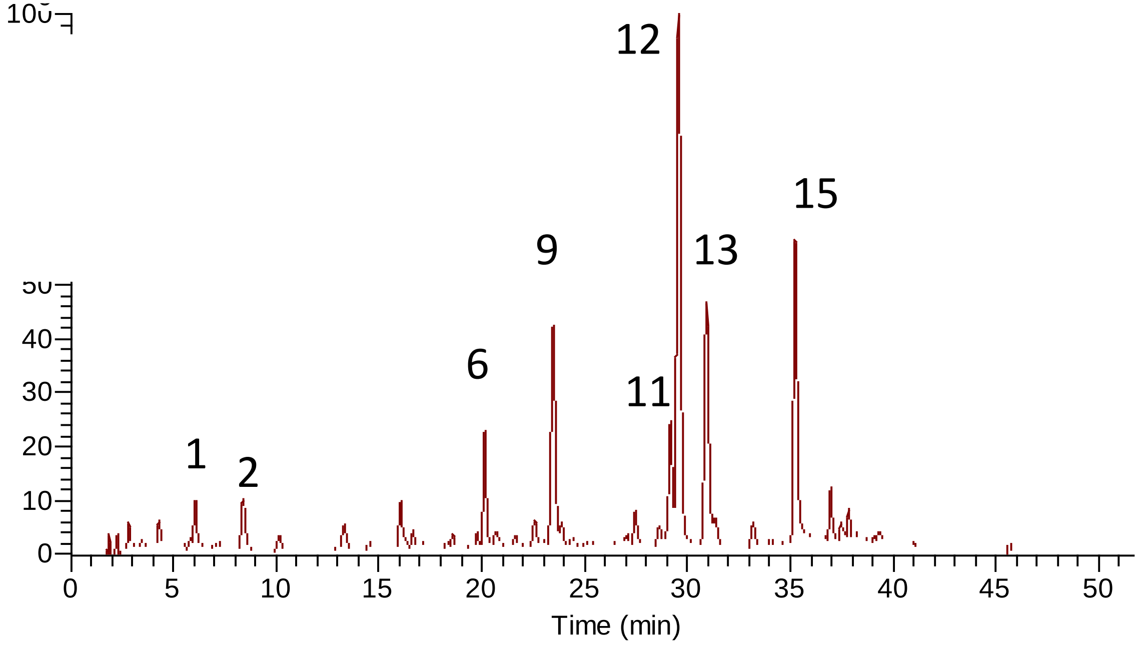

Figure S4. Representative HPLC-PDA-MS chromatogram of the aqueous phase of *Satureja pilosa*. Experimental conditions: column: Zorbax SBAq RP-C18 (150 x 3.0 mm), particle size of 3.5μm (Agilent) at 30 °C. Compounds detected: 12-hydroxyjasmonic acid glucoside (**1**); vicenin 2 (**2**); luteolin-7-*O*-glucoside (**6**); rosmarinic acid (**9**); depside tetramer – not identified (**11**); melitric acid A (**12**); clinopodic acid I (**13**); clinopodic acid O (**15**).

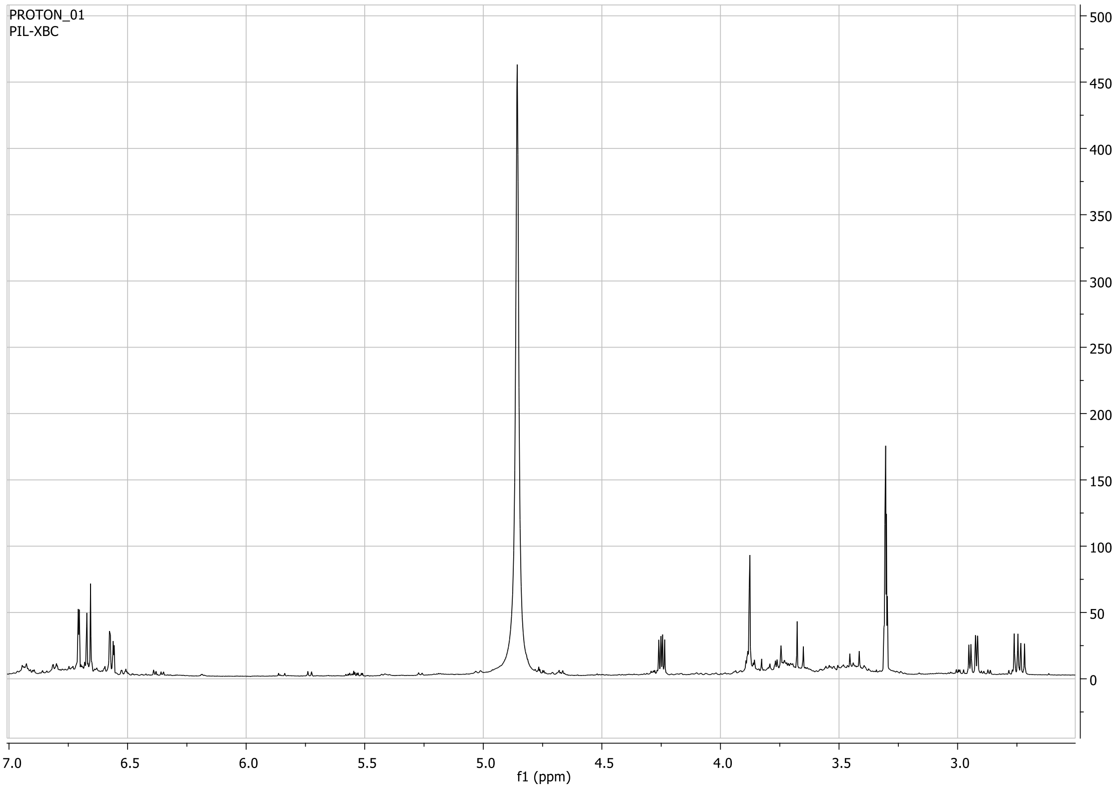

Figure S5. ^{1}H-NMR spectrum (CD_3OD, 500 MHz) of 3,4-dihydroxyphenyllactic acid.

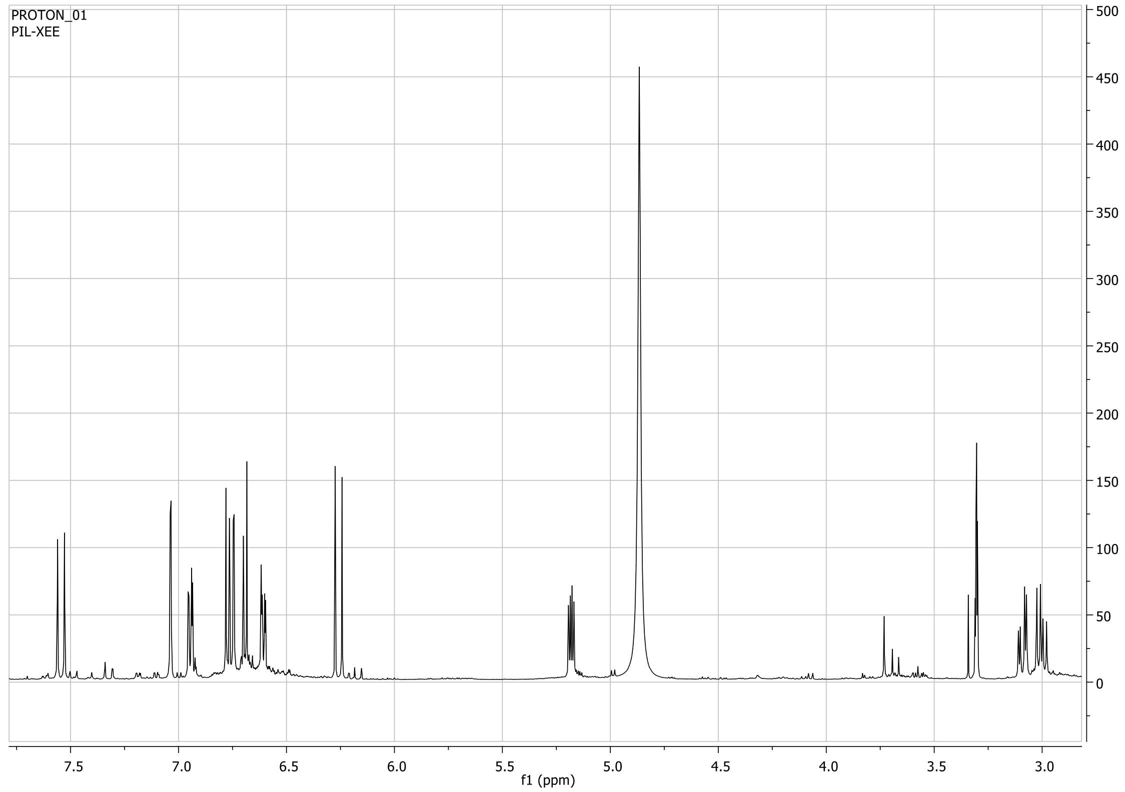

Figure S6. ^{1}H-NMR spectrum (CD_3OD, 500 MHz) of rosmarinic acid.

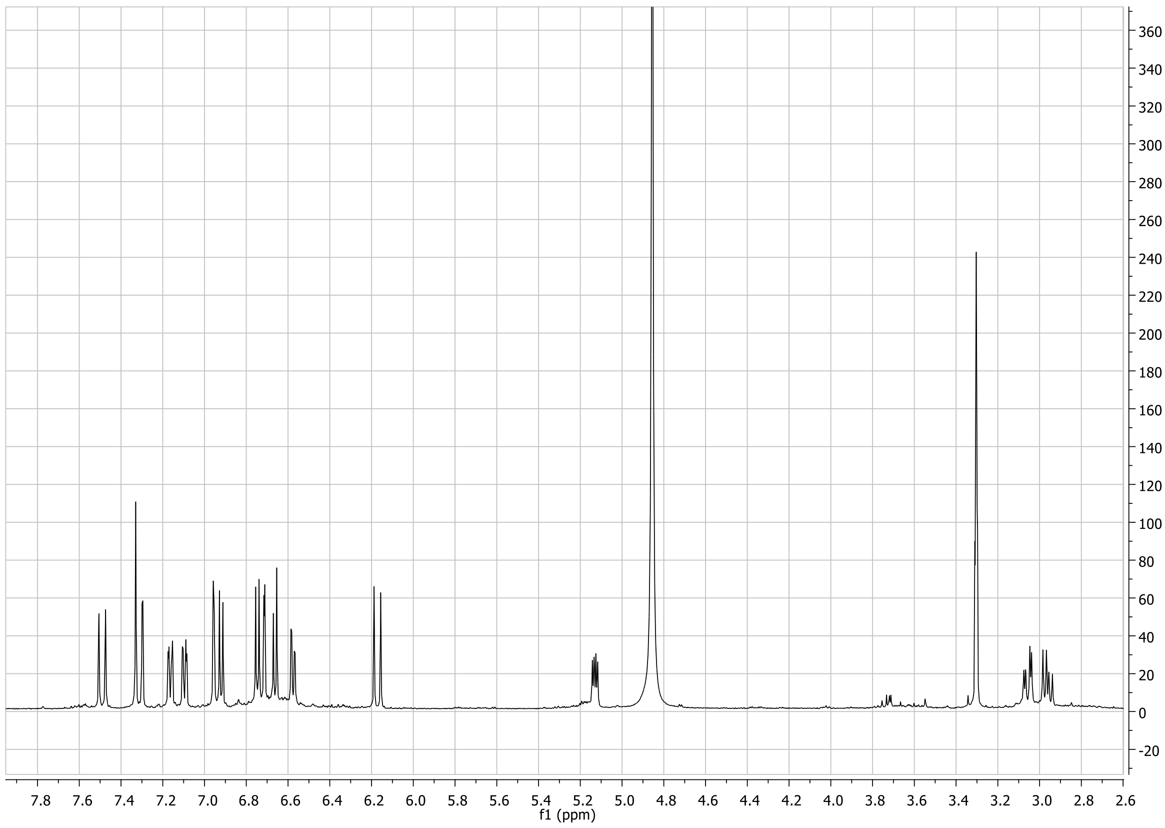

Figure S7. ^{1}H-NMR spectrum (CD_3OD, 500 MHz) of melitric acid A.

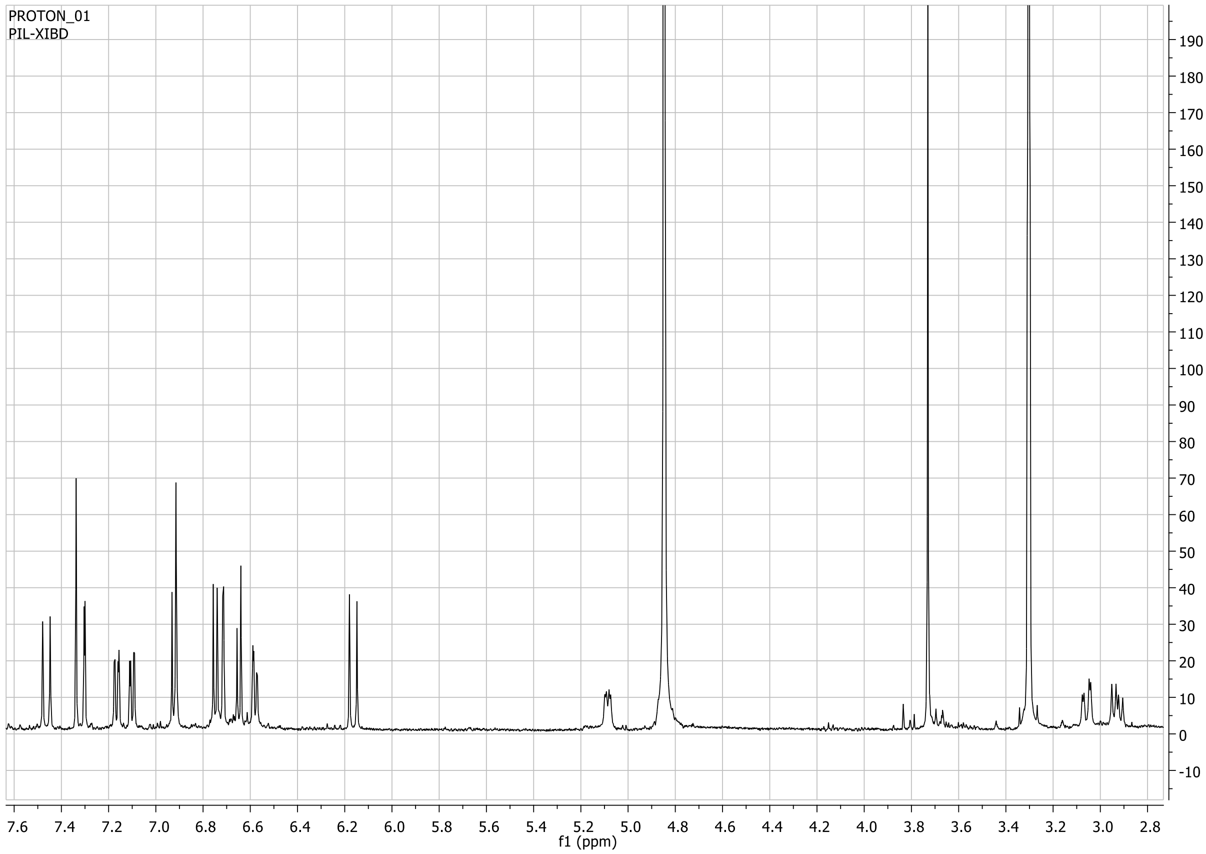

Figure S8. ^{1}H-NMR spectrum (CD_3OD, 500 MHz) of melitric acid A methylester.

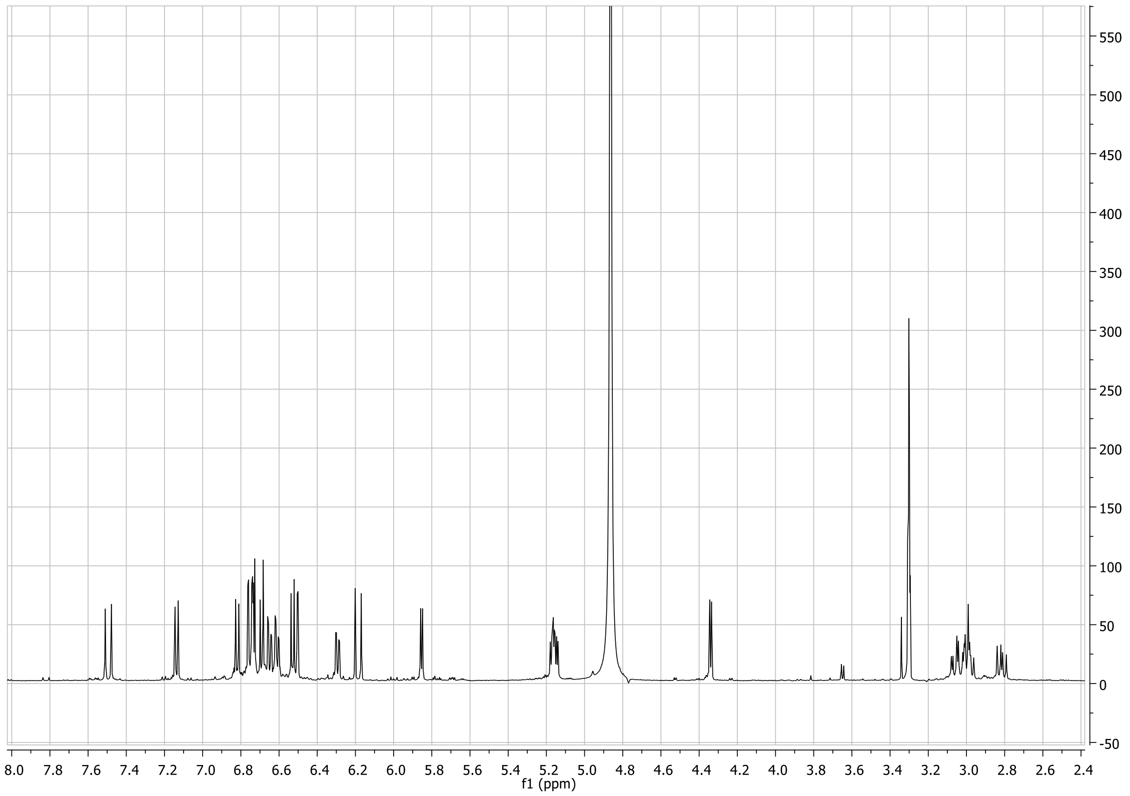

Figure S9. ^{1}H-NMR spectrum (CD_3OD, 500 MHz) of Clinopodic acid I.

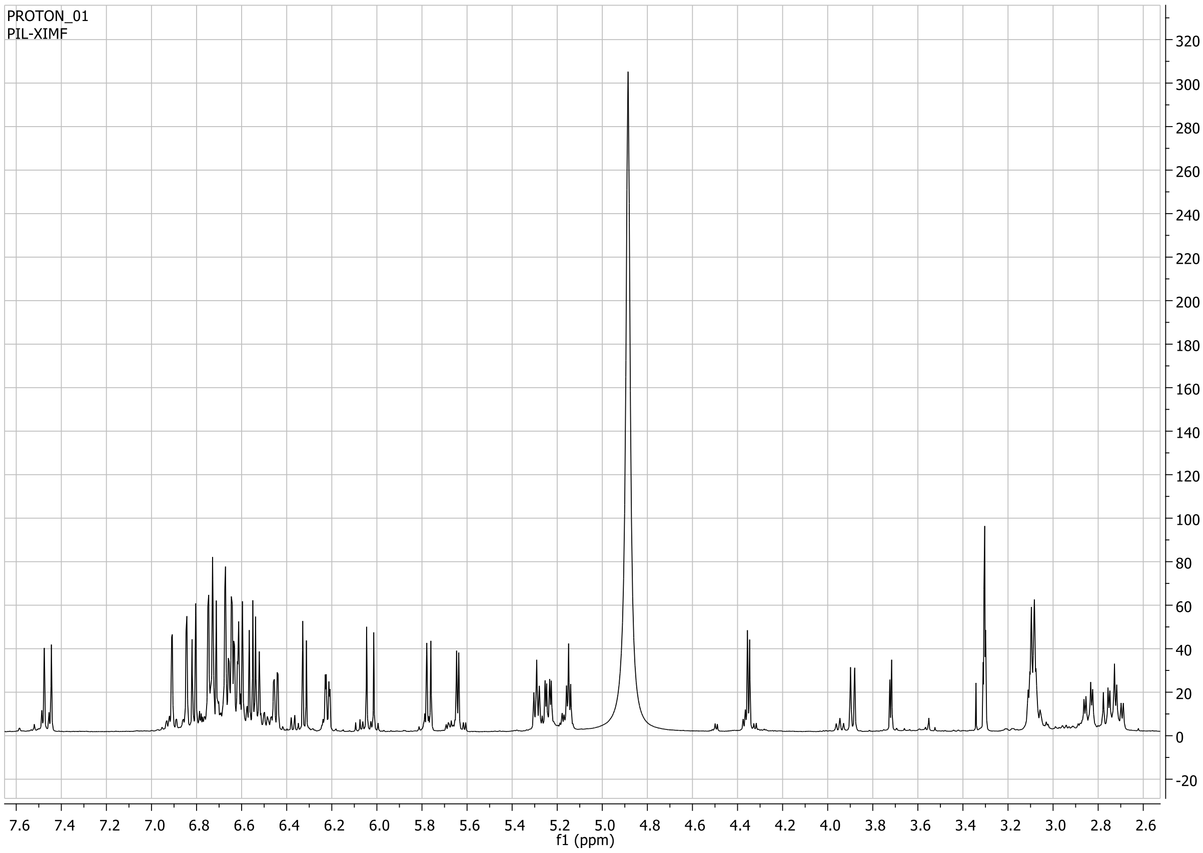

Figure S10. ¹H-NMR spectrum (CD_3OD, 500 MHz) of Clinopodic acid O.

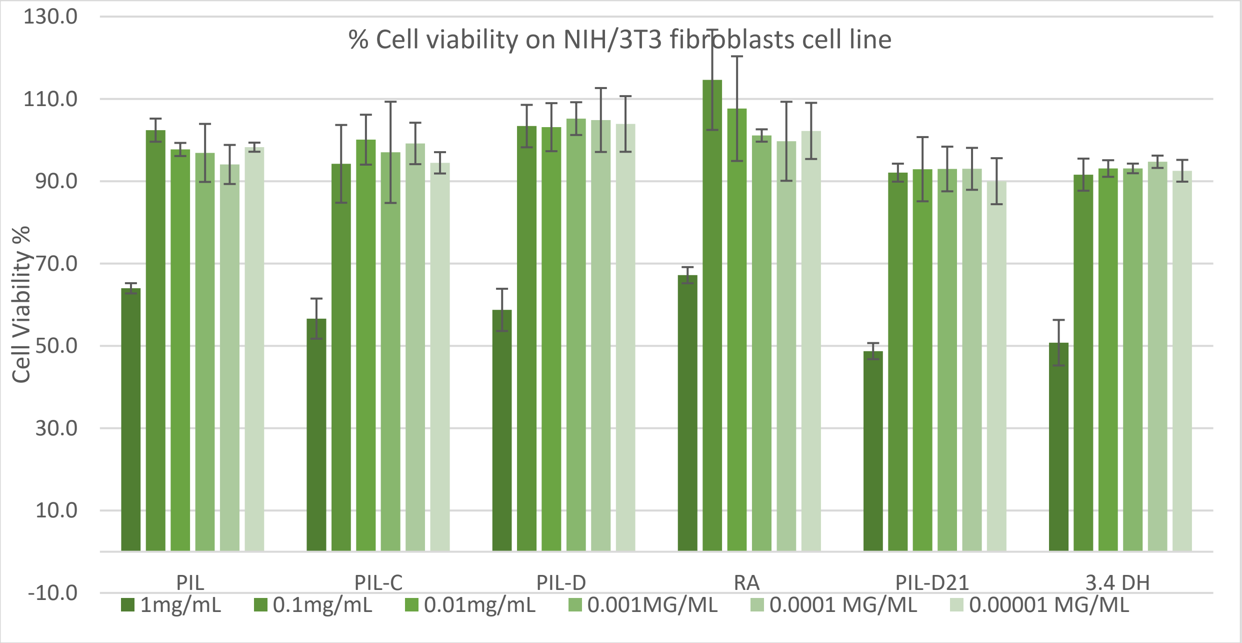

Figure S11. The effects of different extracts of *S. pilosa*, rosmarinic acid, 3,4-dihydrophenyllactic acid and the mixture of clinopodic acid I/melitric acid A on cell viability of 3T3 fibroblasts.

Table S1. Antibacterial activity of representative compounds from *Satureja pilosa Staphylococcus aureus* and *Pseudomonas aeruginosa* expressed as MIC value.

		S. aureus	***P. aeruginosa***
**Sample**		MIC (μg/mL)	
**3**	3,4-dihydroxyphenyllactic acid	>300	>300
**9**	rosmarinic acid	>300	>300
**15**	clinopodic acid O	>300	>300
**13**	clinopodic acid I	>300	>300
**12-13**	PILD-21 (mixture 1:1 of clinopodic acid I and melitric acid A)	>300	>300
PIL	*S. pilosa* initial (methanol)	>300	>300
PIL-C	*S. pilosa* (*n*-BuOH extract)	>300	>300
PIL-D	*S. pilosa* (aqueous extract)	>300	>300
vancomycin		4	-
cirprofloxacin		-	2

Standard: vancomycin (Sigma-Aldrich, Germany) for *S. aureus* and ciprofloxacin (Sigma-Aldrich, Germany) for *P. aeruginosa*.
